# mRNA m^6^A regulates gene expression via H3K4me3 shift in 5’ UTR

**DOI:** 10.1186/s13059-025-03515-8

**Published:** 2025-03-12

**Authors:** Yuna Yang, Yuqing Huang, Tian Wang, Song Li, Jiafu Jiang, Sumei Chen, Fadi Chen, Likai Wang

**Affiliations:** 1 State Key Laboratory of Crop Genetics & Germplasm Enhancement and Utilization, Key Laboratory of Flower Biology and Germplasm Innovation, Ministry of Agriculture and Rural Affairs, College of Horticulture, Nanjing Agricultural University, Nanjing, 210095 P.R. China; 2 Zhongshan Biological Breeding Laboratory, No.50 Zhongling Street, Nanjing, Jiangsu 210014 China

## Abstract

**Background:**

N^6^-methyladenosine (m^6^A) is a prevalent and conserved RNA modification in eukaryotes. While its roles in the 3’ untranslated regions (3’ UTR) are well-studied, its role in the 5' UTR and its relationship with histone modifications remain underexplored.

**Results:**

We demonstrate that m^6^A methylation in the 5’ UTR of mRNA triggers a downstream shift in H3K4me3 modification. This regulatory mechanism is conserved in *Arabidopsis*, rice, and chrysanthemum. The observed shift in H3K4me3 is genetically controlled by m^6^A modifiers and influences gene expression. MTA, the m^6^A methylase, preferentially binds to phosphorylated serine 5 (Ser5P)-CTD of RNA Pol II during transcription, leading to the displacement of ATX1, the H3K4me3 methylase. This dynamic binding of MTA and ATX1 to RNA Pol II ultimately results in the shift of H3K4me3 modification. Genetic evidence demonstrates that m^6^A in the 5' UTR controls H3K4me3 shift, thereby affecting *SEDOHEPTULOSE-BISPHOSPHATASE* expression and leaf senescence.

**Conclusions:**

Our study provides new insights into the roles of m^6^A modification and its crosstalk with histone modification in 5’ UTRs, shedding light on the mechanism of m^6^A-mediated gene expression regulation.

**Supplementary Information:**

The online version contains supplementary material available at 10.1186/s13059-025-03515-8.

## Background

N^6^-methyladenosine (m^6^A) is the most common, abundant, and conserved internal transcriptional modification in eukaryotes, and it is closely related to various biological functions, and plays vital roles in cells [1, 2]. m^6^A is a key regulatory mechanism that controls gene expression, and has been identified in many organisms, including mammals [1], yeast [3], and plants [4]. The “writer” complex installs the mRNA m^6^A modification, a conserved m^6^A methyltransferase complex including methyltransferases like 3 (METTL3), METTL14, Wilms’ tumor 1-associating protein (WTAP), MTA, FIP37 and FIONA1 [4–6]. In addition, the “reader” proteins, such as the YTH domain family, recognize the m^6^A modified transcripts and mediate RNA metabolism [7, 8]. The mRNA m^6^A modification is reversible and can be removed by “eraser” proteins, including fat-mass and obesity-associated protein (FTO), alkylated DNA repair protein alkB homolog 5 (ALKBH5) [9, 10], ALKBH9B [11] and ALKBH10B [12].

The roles and regulatory mechanisms of m^6^A modification in 3’ untranslated regions (3’ UTR) have been extensively studied. However, little attention has been given to 5′ UTR-specific m^6^A functions. Recent studies showed that m^6^A modifications in 5’ UTR regions are essential in regulating cortical development in mice, especially through mRNA transport and surveillance [13]. mRNAs containing m^6^A in their 5’ UTR can be translated cap-independently, by binding eukaryotic initiation factor 3 (eIF3) to recruit the 43S complex to initiate translation without the cap-binding factor [14]. 5′ UTR-specific m^6^A controls ribosome scanning and subsequent start codon selection to control global alternative translation during amino acid starvation [15]. In response to heat shock stress, m^6^A is preferentially deposited at the 5′UTR of newly transcribed mRNAs, resulting from stress-induced nuclear localization of m^6^A “reader” YTHDF2 [16]. All previous studies have focused on the roles of 5′ UTR-specific m^6^A in post-transcriptional regulation. However, whether these m^6^A modifications are involved in transcriptional regulation, especially in plant species, remains unknown.

Chromatin states regulate gene expression and responses to environmental stimuli during plant development. Various histone modification combinations, including histone methylation, acetylation, and phosphorylation, determine chromatin states. Recent studies have shown that m^6^A regulates chromatin accessibility and gene transcription through DNA demethylation [17], chromosome-associated regulatory RNAs (carRNAs) [18] and histone modification [19]. In addition, the m^6^A modification of mRNAs is mainly guided by the tri-methylation of histone H3 Lys-36 (H3K36me3) in animals [20]. The loss of H3K36me3 leads to a significant reduction in RNA m^6^A modification [20]. However, in plants, the histone modification H3K36me2, not H3K36me3, is a potential determinant of m^6^A modification [21]. Many other histone modification markers are associated with m^6^A methylation. For instance, H3K4me3 histone modifications are typically associated with active gene promoters. In mammalian research, it has been shown that the RNA m^6^A methyltransferase METTL3 binds to the transcription start site (TSS) in the vicinity of H3K4me3 histone marker peaks and facilitates m^6^A modification and enhances translation of the associated RNA transcript [22]. H3K27me3 histone modification, unlike H3K4me3, typically inhibits gene expression within heterochromatin. Although over-representation of the H3K27me3 histone modification near the m^6^A peaks was not detected [20], METTL3 depletion was observed to reduce the levels of H3K27me3 in animals [23]. However, the link between histone modifications and RNA m^6^A methylation remains unclear in plant.

In this work, by analyzing m^6^A methylation in different regions, we observed a consistent and conserved association between m^6^A methylation specifically in the 5’ UTR regions, referred to as m^6^A_5_, and low levels of transcription across multiple plant species, including *Arabidopsis*, rice, and chrysanthemum. We identified that H3K4me3 modification was involved in the m^6^A-associated expression regulation of m^6^A_5_. We found that MTA preferentially binding to the phosphorylated serine 5 (Ser5P)-CTD domain of RNA Pol II displaces the H3K4me3 methylase ATX1, resulting in H3K4me3 modification shifts at the transcription initiation and early elongation process. Through genetic validation, we observed that the shift of H3K4me3 modification is regulated by m^6^A modification in 5’ UTR regions, resulting in gene expression and leaf senescence regulation. Overall, this work establishes a mechanism by which m^6^A modification in 5’ UTR regions regulates H3K4me3 shift to finetune gene expression.

## Results

### m^6^A methylation, uniquely deposited in 5’ UTR regions, contributes to gene repression

**Fig. 1:**
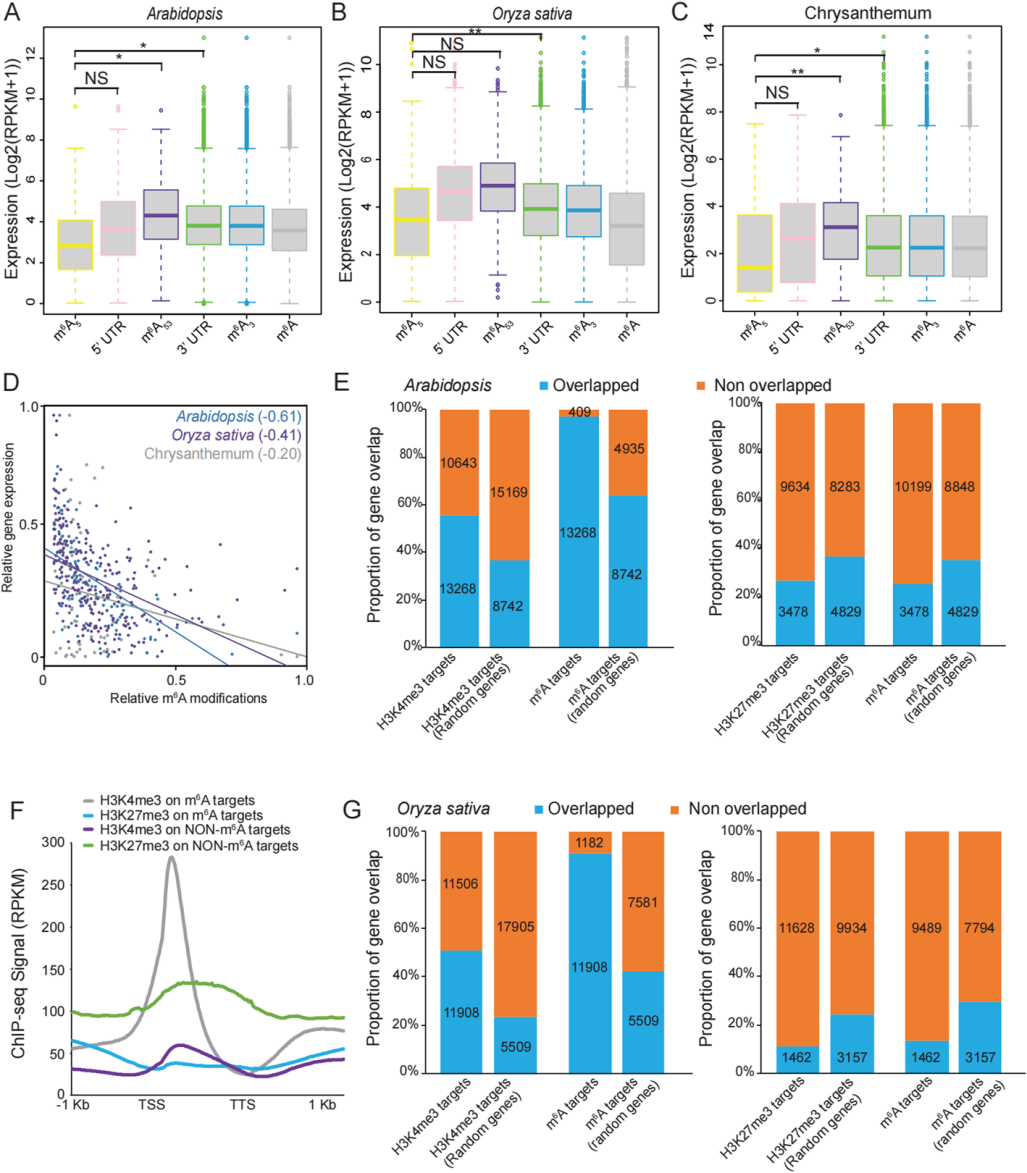
m^6^A methylation depositing in 5’ UTR regions represses gene expression. **A-C** Boxplot show the comparison of gene expression among genes with m^6^A modification at different regions in *Arabidopsis*, *Oryza sativa* and chrysanthemum*.* 5’ UTR: genes with m^6^A methylation in 5’ UTR region; m^6^A_53_: genes with m^6^A methylation in both 5’ UTR and 3’ UTR region; m^6^A_5_: genes with m^6^A methylation only in 5’ UTR region; m^6^A_3_: genes with m^6^A methylation only in 3’ UTR region; 3’ UTR: genes with m^6^A methylation in 3’ UTR region; m^6^A: all genes with m^6^A methylation. Statistical analysis was conducted using the Student’s *t*-test. ** indicates P value < 0.01, * indicates P value < 0.05, and NS indicates no significance. **D** Relationship between m^6^A modification and target gene expression in *Arabidopsis*, *Oryza sativa* and chrysanthemum. Scatter plots showing the correlation between m^6^A fold enrichment and target mRNA abundance. **E** Comparison of H3K4me3-marked genes or H3K27me3-marked genes with genes marked by m^6^A in *Arabidopsis*. **F** Distribution of H3K4me3 or H3K27me3 reads in transcript segments with or without m^6^A modification. **G** Comparison of H3K4me3-marked genes or H3K27me3-marked genes with genes marked by m^6^A in *Oryza sativa*

The untranslated regions (UTRs) are recognized for their important role in regulating gene expression [24]. To explore the potential roles of 5’ UTRs in m^6^A-mediated transcriptional regulation, we re-analyzed the publicly available meRIP-seq and RNA-seq data in *Arabidopsis* from NCBI GEO GSE180768. We divided genes into five groups based on the location of m^6^A methylation in the gene transcripts in *Arabidopsis*, including genes with m^6^A methylation in 5’ UTR region (5’ UTR), genes with m^6^A methylation in both 5’ UTR and 3’ UTR region (m^6^A_53_; Additional file 1: Fig. S1A), genes with m^6^A methylation only in 5’ UTR region (m^6^A_5_; Additional file 1: Fig. S1B), genes with m^6^A methylation only in 3’ UTR region (m^6^A_3_; Additional file 1: Fig. S1C), and genes with m^6^A methylation in 3’ UTR region (3’ UTR). Previous studies have consistently reported lower levels of m^6^A modification in the 5' UTR region compared to the 3' UTR region [25, 26]. To validate the presence of m^6^A modification signals within the 5' UTR regions, we conducted a re-analysis of publicly available meRIP-seq data obtained from *Arabidopsis* plants harboring mutations in FIP37, a known m^6^A writer protein. The dataset was retrieved from the NCBI GEO GSE174573. Our analysis revealed that ~ 94% of the 5’ UTR genes with m^6^A modification identified in Col-0 were absent in the meRIP-seq data of *fip37* mutant (Additional file 1: Fig. S1D). This observation implies that the majority of genes with m^6^A modification in their 5’ UTR regions lost this modification in the mutants, indicating the reliability of these 5’ UTR genes with m^6^A modification. We then compared their expression with all m^6^A-methylated genes (m^6^A). We observed that m^6^A_5_ genes showed the lowest expression, compared to genes in other groups (Fig. 1A), indicating that the 5’ UTR-specific m^6^A modification is associated with low transcription levels.

To investigate whether m^6^A_5_-associated low levels of transcription are evolutionarily conserved, we re-analyzed publicly available meRIP-seq and RNA-seq data from NCBI GEO GSE211828 in panicles of rice, a model crop plant, and performed meRIP-seq using a commercial anti-m^6^A antibody (202–003; Synaptic Systems) in leaf tissues of chrysanthemum, a segmental allopolyploid, self-incompatible and highly heterozygous plant [27] (Additional file 2: Table S1). We further divided the genes into five groups based on the location of m^6^A methylation in the gene transcripts in rice and chrysanthemum and then compared the group expression levels. Consistent with the results obtained in *Arabidopsis*, m^6^A_5_ showed the lowest expression in rice and chrysanthemum (Fig. 1B and C). These results indicate that m^6^A methylation, uniquely deposited in 5’ UTR regions, is associated with lower gene expression.

To analyze the relationship between m^6^A modification and gene expression in m^6^A_5_ genes, we performed a correlation analysis between m^6^A peak fold-enrichment and target mRNA abundance. The negative correlations were conservatively observed in *Arabidopsis*, rice and chrysanthemum (Fig. 1D), indicating that m^6^A methylation uniquely depositing in 5’ UTR regions contributes to gene repression.

### H3K4me3 is involved in the transcriptional regulation of m^6^A-modified genes

Chromatin remodeling plays an essential role in transcriptional regulation by altering the chromatin architecture to allow dynamic access to condensed DNA. Recent studies have shown that the chromatin state is regulated by m^6^A methylation of chromosome-associated regulatory RNA or by the methyltransferase METTL3/METTL14 [18, 28], indicating a direct link between m^6^A modification and the dynamic chromatin state. Histone H3 Lys 4 tri-methylation (H3K4me3) is an evolutionarily conserved epigenetic marker of transcriptionally active euchromatin [29], and H3K27me3 contributes to the repression of its associated genes [30]. To determine whether these two epigenetic histone markers correlate with levels of m^6^A modification in mRNA, we re-analyzed the H3K4me3 and H3K27me3 dataset from NCBI GEO GSE193251 [31]. We compared the genes associated with H3K4me3 and H3K27me3 with m^6^A-modified transcripts. As a control, we also included randomly generated genes with the same number of H3K4me3 targets. Our analysis revealed that approximately 60% of genes marked with H3K4me3 exhibited m^6^A modifications, accounting for 95% of the total m^6^A-modified genes (Fig. 1E, Additional file 1: Fig. S1E). In contrast, only 40% of randomly generated genes carried m^6^A modifications, accounting for 60% of the total m^6^A-modified genes (Fig. 1E). Interestingly, only 26% of genes marked with H3K27me3 overlapped with m^6^A-modified transcripts, representing 25% of the total m^6^A-modified genes. This proportion was lower than the overlapping proportion observed with randomly generated genes (Fig. 1E). To further validate the enrichment of both histone marks on m^6^A-modified genes, we examined the H3K4me3 and H3K27me3 profiles around the gene body of m^6^A-modified genes and compared them with genes without m^6^A modification. We observed a significant enrichment of H3K4me3 in the vicinity of m^6^A targets, while minimal enrichment was observed in genes lacking m^6^A modification (Fig. 1F). Additionally, we found a relatively higher level of H3K27me3 modification in genes without m^6^A modification compared to genes with m^6^A modification (Fig. 1F). Collectively, these findings suggest that H3K4me3 is involved in the transcriptional regulation of m^6^A-modified genes.

To investigate whether the co-enrichment of H3K4me3 and m^6^A modification exists in other plant species, we obtained H3K4me3 and H3K27me3 ChIP-seq data from the NCBI GEO GSE142570 [32] for rice panicles. Consistent with the findings in *Arabidopsis*, our analysis revealed that H3K4me3, but not H3K27me3, exhibited a specific enrichment in m^6^A-modified genes (Fig. 1G, Additional file 1: Fig. S1F and G). These results provide further support for the presence of co-enrichment between H3K4me3 and m^6^A modification in multiple plant species. The high percentage of m^6^A-modified genes among those marked with H3K4me3 and the specific enrichment of H3K4me3 in m^6^A-modified genes reinforce the association between H3K4me3 and the regulation of m^6^A-modified mRNA transcripts in a conservative manner across different plant species.

### m^6^A depositing in 5’ UTR shifts H3K4me3 modification

**Fig. 2:**
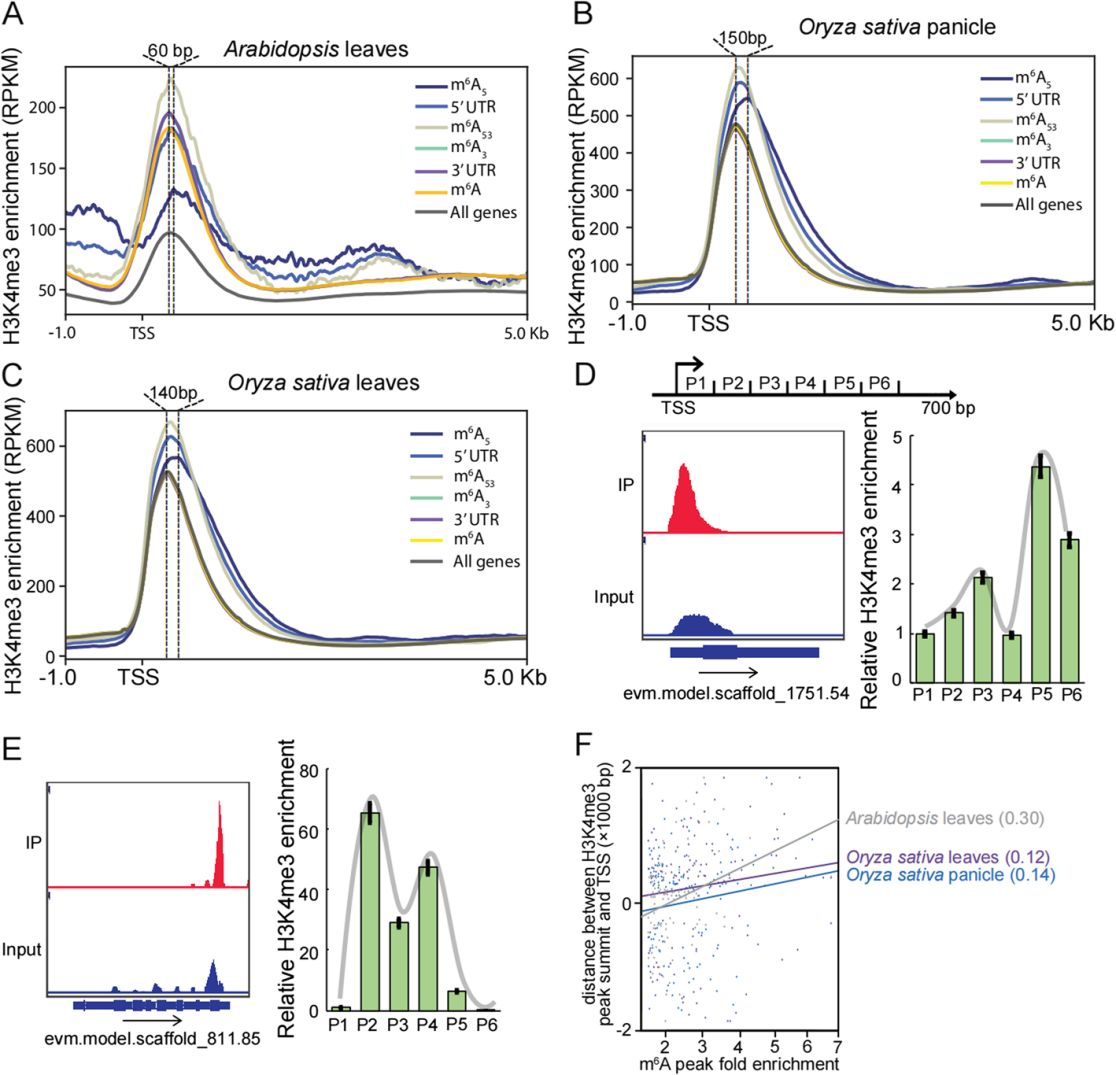
The relationship between H3K4me3 and m^6^A methylation. **A-C** The meta-gene profiles of the H3K4me3 generated along gene bodies in leaves of *Arabidopsis* (**A**), panicle (**B**) and leaves (**C**) of *Oryza sativa*. 5’ UTR: genes with m^6^A methylation in 5’ UTR region; m^6^A_53_: genes with m^6^A methylation in both 5’ UTR and 3’ UTR region; m^6^A_5_: genes with m^6^A methylation only in 5’ UTR region; m^6^A_3_: genes with m^6^A methylation only in 3’ UTR region; 3’ UTR: genes with m^6^A methylation in 3’ UTR region; All genes: all the genes in corresponding genome that have both 5’ UTR and 3’ UTR; m^6^A: genes with m^6^A methylation. **D-E** Genome browser traces and ChIP-qPCR to show the m^6^A enrichment and H3K4me3 modification, respectively, for genes with m^6^A modification specifically in 5’ UTR regions (**D**) and 3’ UTR regions (**E**). About 700 bp regions were divided into 6 parts for ChIP-qPCR examination. **F** Correlation analysis to examine the relationship between m^6^A modification and the shift of H3K4me3 in the 5’ UTR regions of m^6^A_5_ genes. We obtained the fold enrichment of each m^6^A peak and calculated the distance between the summit of H3K4me3 peak and the TSS. Subsequently, we calculated the Pearson correlation coefficient (PCC) to assess the correlation between these variables

To explore the combined roles of epigenetic histone markers and mRNA m^6^A methylation in transcriptional regulation, we examined the H3K4me3 profiles around the TSS region of different gene groups. In *Arabidopsi*s*,* we observed low levels of H3K4me3 modification in m^6^A_5_ genes (Fig. 2A), which is consistent with the low expression of these genes (Fig. 1A). To our surprise, we observed a difference in the location of H3K4me3 enrichment peaks, relative to the TSS, between genes with m^6^A modification in 5’ UTR regions (m^6^A_5_, m^6^A_53_ and 5’ UTR genes) and m^6^A_3_ genes (Fig. 2A). To determine the exact length of this peak shift, we used 10-bp resolution intervals (10-bp bins) to partition the sequences downstream of the TSS and counted the number of bins between the TSS and H3K4me3-enriched peak summits in the m^6^A_3_ and m^6^A_5_ genes. We observed that the shift of the H3K4me3 peak on the m^6^A_5_ genes was 60 bp (from 310 to 370 bp downstream of the TSS; Additional file 1: Fig. S2A) compared to that in the m^6^A_3_ genes.

To examine whether the H3K4me3 modification shifts at m^6^A_5_ genes generally appeared in other species, we examined the H3K4me3 profiles around the TSS region of different gene groups in panicles of rice and identified that the H3K4me3 modification shifted 150 bp downstream of TSS when m^6^A methylation deposited in the 5’ UTR of mRNA, compared with these deposited in 3’ UTR (Fig. 2B, Additional file 1: Fig. S2B). To explore whether this downstream shift is observed in other rice tissues, we re-analyzed meRIP-seq data from NCBI GEO GSE211828 [33] and H3K4me3 ChIP-seq data from NCBI GEO GSE142570 in rice leaves [32] and observed a 140 bp downstream shift in m^6^A_5_ genes (Fig. 2C and Fig. S2C). We confirmed that a downstream shift in H3K4me3 enrichment in m^6^A_5_ occurred in the chrysanthemum leaf tissues (Fig. 2D-E and Additional file 1: Fig. S2D-H). These data suggest that H3K4me3 modification shifts in m^6^A_5_ genes occur in different tissues and plant species.

To examine the relationship between m^6^A modification and the shift of H3K4me3 in the 5’ UTR regions of m^6^A_5_ genes, we conducted a comparison of m^6^A enrichment with the downstream distance between the summit of the H3K4me3 peak and the TSS of target genes. The results demonstrated a positive correlation between higher m^6^A modification levels and larger downstream shifts of H3K4me3 enrichment in both *Arabidopsis* and rice (Fig. 2F), indicating that the shift of H3K4me3 modification in the 5' UTR regions of m^6^A_5_ genes is indeed correlated with m^6^A modification.

### The downstream shift of H3K4me3 is genetically affected by the m^6^A writer and eraser

The m^6^A_53_ genes exhibit strong m^6^A modifications in the 3’ UTR regions, which may play dominant roles in gene expression regulation and overshadow the significance of 5’ UTR regions. Therefore, our focus was specifically on genes that possess m^6^A modification exclusively in their 5’ UTR regions (referred to as m^6^A_5_ genes).

**Fig. 3:**
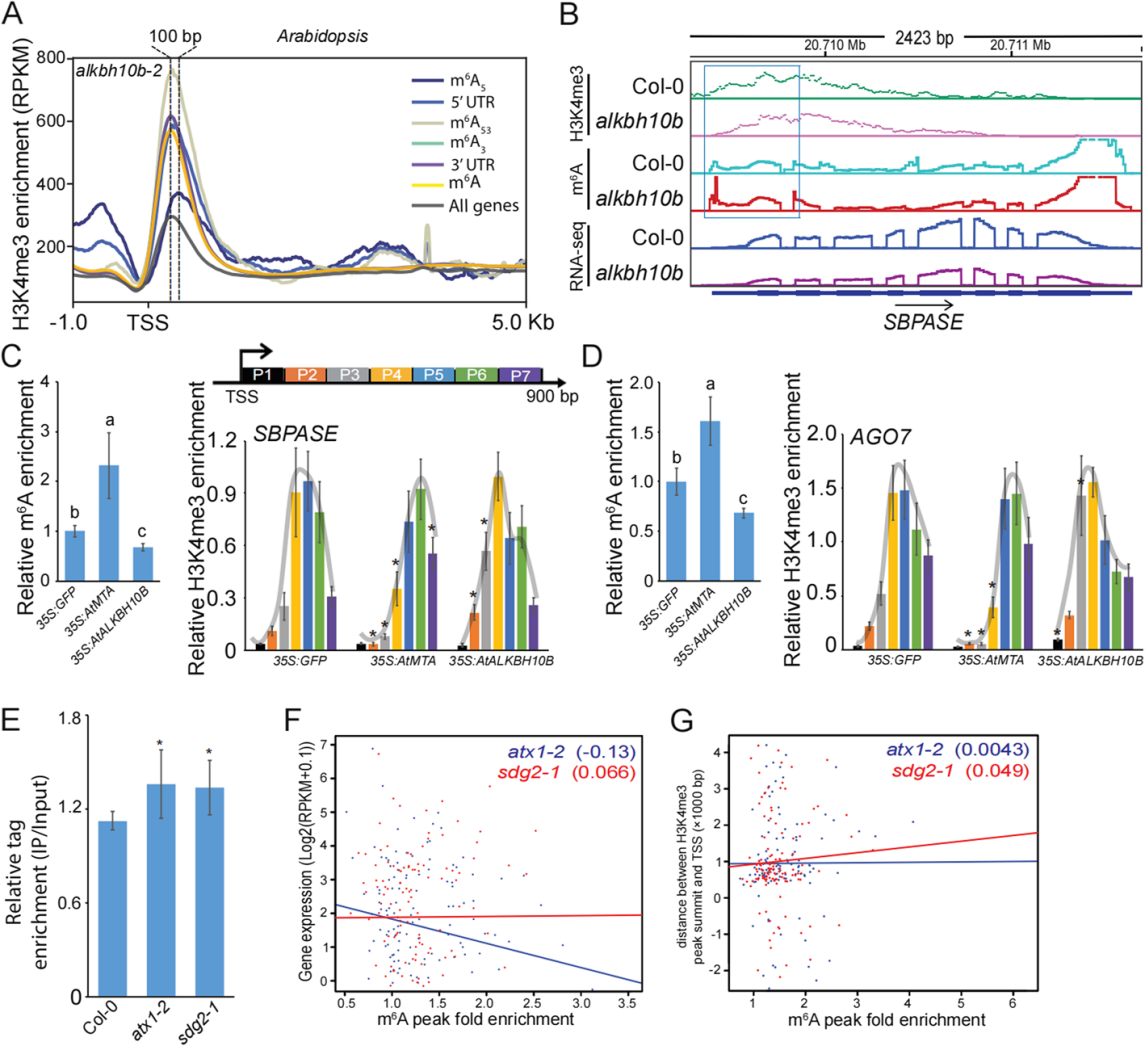
The downstream shift of H3K4me3 is genetically affected by the m^6^A writer and eraser. **A** The meta-gene profiles of the H3K4me3 generated along gene bodies in leaves of *Arabidopsis alkbh10b-2* mutant. 5’ UTR: genes with m^6^A methylation in 5’ UTR region; m^6^A_53_: genes with m^6^A methylation in both 5’ UTR and 3’ UTR region; m^6^A_5_: genes with m^6^A methylation only in 5’ UTR region; m^6^A_3_: genes with m^6^A methylation only in 3’ UTR region; 3’ UTR: genes with m^6^A methylation in 3’ UTR region; All genes: all the genes in corresponding genome that have both 5’ UTR and 3’ UTR; m^6^A: genes with m^6^A methylation. **B** Screenshots showing normalized sequencing signals of H3K4me3, meRIP-seq and RNA-seq in both Col-0 and *alkbh10b* mutant over a representative gene. The binding levels or gene expression levels were indicated by reads per kilobase per million reads in sample (RPKM). The 5’ UTR regions with H3K4me3 shit and elevated m^6^A levels were highlighted with blue box. **C-D** Protoplast transient assay system to examine the m^6^A levels and H3K4me3 shift in *SBPASE* (**C**) and *AGO7* (**D**) genes. The protoplasts were transformed with *35S:AtMTA-GFP*, *35S:AtALKBH10B* or empty vectors. We assessed m^6^A modification enrichment (left panel) and H3K4me3 enrichment (right panel) on each gene. Trendlines were added to trace the H3K4me3 shift. Different letters were used to indicate statistically significance difference (*P* < 0.05, Student’s t-test). Diagram in (**C**) illustrates the primers used for H3K4me3 ChIP-qPCR. **E** Comparison of m^6^A modification in 5’ UTR regions among Col-0, *atx1-2* and *sdg2-1* plants. * indicates *P* value < 0.05. **F-G** Correlation analysis to examine the relationship between m^6^A modification and gene expression (**F**), and the relationship between m^6^A modification and H3K4me3 downstream shift (**G**) in m^6^A_5_ genes in *atx1-2* and *sdg2-1*. The Pearson Correlation Coefficient (PCC) was used to assess the correlation between these variables

To investigate the impact of m^6^A demethylases on H3K4me3 modification shifts, we utilized the *alkbh10b-2* mutant (Salk_107289C) [34]. This mutant contains a T-DNA insertion in the coding gene *ALKBH10B*, which is responsible for mRNA m^6^A erasure (Additional file 1: Fig. S3A). Consequently, the mutant of *ALKBH10B* exhibits a global increase in m^6^A modification (Additional file 1: Fig. S3B) [12, 34]. We performed ChIP-seq using an H3K4me3 antibody. In the *alkbh10b*−2 mutant, we observed a 100 bp downstream shift of H3K4me3 in the m^6^A_5_ genes (Fig. 3A and B). This shift was more prominent than that observed in the Col-0 wild-type plants (Fig. 2A), providing evidence for the potential role of m^6^A demethylases in regulating the downstream shift of H3K4me3.

To validate the impact of ALKBH10B-mediated m^6^A modification on H3K4me3 shift, we performed ChIP-qPCR using an anti-H3K4me3 antibody and m^6^A-qPCR using an anti-m^6^A antibody in *Arabidopsis* protoplast cells that were transformed with *35S:AtALKBH10B-GFP*. These results were compared with cells transformed with empty vectors. The data indicated decreased m^6^A modification levels in the 5’ UTR regions upon transformation with *35S:AtALKBH10B-GFP* (Fig. 3C and D, Additional file 1: Fig. S3C), while H3K4me3 modification in the 5’ UTR regions closer to the TSS (Fig. 3C and D), consistent with the observations in the *alkbh10b-2* mutant (Fig. 3A and B).

Next, we employed protoplast transient assay system to examine whether MTA-mediated m^6^A modification regulates the shift of H3K4me3. We observed increased m^6^A modification levels in the 5’ UTR regions upon transformation with *35S:AtMTA-GFP* (Fig. 3C and D), whereas H3K4me3 modification in the 5’ UTR regions more distant from the TSS compared to cells transformed with empty vectors (Fig. 3C and D), indicating that the downstream shift of H3K4me3 is affected by the m^6^A writer and eraser.

### m^6^A-mediated gene expression depends on H3K4me3 modification in 5’ UTR regions

To investigate whether H3K4me3 modification regulates m^6^A levels in the 5’ UTR regions, we performed meRIP-seq analysis using leaves from *atx1-2* (Salk_149002C) [35] and *sdg2-1* (Salk_021008) [36] mutants (Fig. S3) that carry T-DNA insertions in the *SET DOMAIN GROUP* (*SDG*) genes, which have been previously shown to be essential for H3K4 trimethylation. We quantified the m^6^A enrichment in the 5’ UTR regions of m^6^A_5_ genes. The results revealed higher m^6^A modification levels in the *atx1-2* and *sdg2-1* mutants compared to the Col-0 control (Fig. 3E), indicating a negative effect of H3K4me3 modification on m^6^A levels.

Next, we examined the gene expression patterns in both mutants, and compared them with the changes in m^6^A modifications. Notably, in the *sdg2-1* mutant, we did not detect a significant correlation between the changes in m^6^A and the variations in gene expression (Fig. 3F). Moreover, in the *atx1-2* mutant, we only observed a weak negative correlation (Fig. 3F), which was significantly lower than that observed in Col-0, the control strain with normal H3K4me3 modification (Fig. 1D). We then examined the correlation between the changes of m^6^A and H3K4me3 modification shifts, we found no significant correlation in *atx1-2* and *sdg2* mutants (Fig. 3G), which was significantly lower than that observed in Col-0 (Fig. 2F). These findings suggest that m^6^A modification alone is insufficient to regulate gene expression in the absence of H3K4me3 modification in the 5’ UTR regions, further indicating that the presence of H3K4me3 modification is crucial for the regulatory effects of m^6^A in the 5' UTR regions.

### MTA and ATX1 interact with CTD of RNA Pol II in vitro and in vivo

**Fig. 4:**
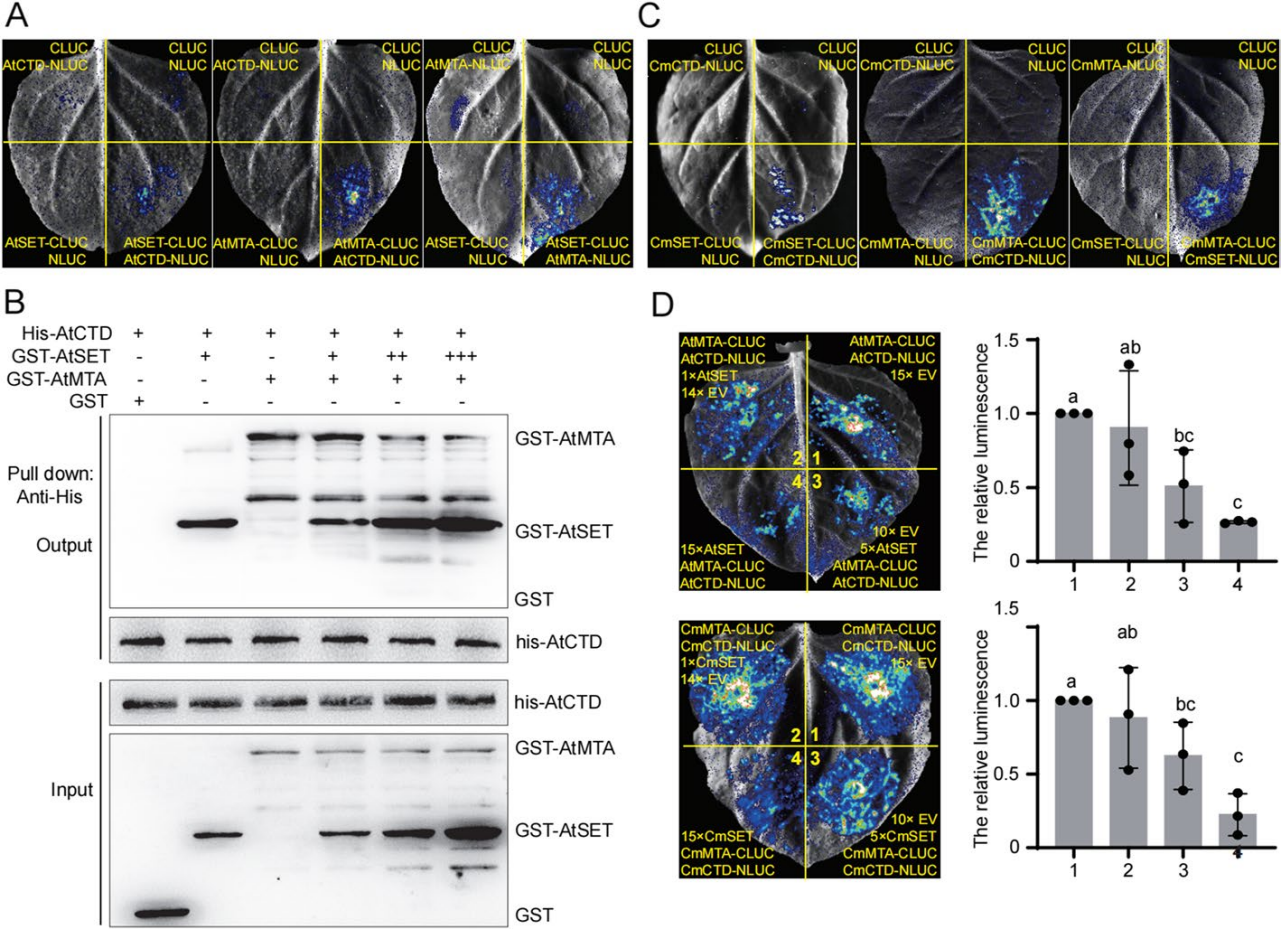
MTA and ATX1 interact with CTD of RNA Pol II. **A** Split-luciferase complementation (Split-LUC) assays examining the interaction between AtSET and AtCTD (left panel), AtMTA and AtCTD (middle panel), and AtSET and AtMTA (right panel). Split-LUC assays were conducted by the infiltration of *Agrobacterium* carrying indicated constructs into *N. benthamiana* leaves. Images of plants sprayed with 500 μM luciferin and placed in the dark for 5 min are shown. **B** Pull-down assays to examine the interaction between AtSET or AtMTA and AtCTD. Increasing concentrations of GST-AtSET was respectively incubated with a fixed concentration of GST-AtMTA. GST-fusion proteins and GST were detected with an anti-GST antibody. Input means total protein lysate without immunoprecipitation. **C** Split-LUC assays examining the interaction between CmSET and CmCTD (left panel), CmMTA and CmCTD (middle panel), and CmSET and CmMTA (right panel). Split-LUC assays were conducted by the infiltration of *Agrobacterium* carrying indicated constructs into *N. benthamiana* leaves. Images of plants sprayed with 500 μM luciferin and placed in the dark for 5 min are shown. **D** Split-LUC assays examining the competition between AtMTA and AtSET (upper panel), and CmMTA and CmSET (lower panel). Split-LUC assays were conducted by the infiltration of *Agrobacterium* carrying indicated constructs into *N. benthamiana* leaves. Images of plants sprayed with 500 μM luciferin and placed in the dark for 5 min are shown. Quantitation of luciferase intensity from three biological replications are shown in plots (error bars are SD). Different letters were used to indicate statistically significance difference (*P* < 0.05, Student’s t-test)

Previous studies have reported that ATX1, the H3K4me3 writer containing a SET domain, binds to the CTD domain of RNA Pol II [37]. Similarly, MTA, the writer of m^6^A, has been shown to interact with RNA Pol II in *Arabidopsis* [38]. Based on these reports, we hypothesized that MTA may compete with ATX1 for binding to RNA Pol II during transcription initiation, leading to a downstream shift in H3K4me3 modification. To test this hypothesis, we performed in vitro and in vivo protein–protein interaction assays, including split-luciferase complementation (Split-LUC) imaging and pulldown assays. In the Split-LUC assay, we cloned the coding sequences of each gene/domain fused with either the C-terminal or N-terminal half of LUC. These constructs were then transformed into *Agrobacterium* strain GV3101 and infiltrated into *Nicotiana benthamiana* leaves. We observed luciferase activity when co-infiltrating AtSET-CLUC and AtCTD-NLUC constructs, indicating interaction between the two proteins (Fig. 4A). Interestingly, we also detected luciferase activity when co-infiltrating the AtMTA-CLUC and AtCTD-NLUC constructs, and AtSET-CLUC and AtMTA-NLUC constructs (Fig. 4A). To further confirm these interactions, we conducted a pull-down assay. In this assay, we cloned AtSET and AtMTA fused with GST and AtCTD fused with a His tag, respectively. We observed bands of the expected molecular weight for GST-AtMTA and GST-AtSET in the pull-down assay using anti-His antibody (Fig. 4B). These interacts were also detected when using the coding sequences from chrysanthemum (Fig. 4C), suggesting that the interactions between ATX1, MTA, and the CTD of RNA Pol II are conserved across plant species.

### MTA competes ATX1 to interact with CTD of RNA Pol II in vitro and in vivo

To explore the competitive binding between ATX1 and MTA to the CTD of RNA Pol II, we co-infiltrated *Nicotiana benthamiana* leaves with AtMTA-CLUC and AtCTD-NLUC constructs, along with varying amounts of AtSET or an empty vector (EV) control. As the concentration of AtSET increased, the luciferase activity, indicative of the interaction between AtMTA and AtCTD, significantly decreased (Fig. 4D, upper panel). Similar results were observed when using genes from chrysanthemum (Fig. 4D, lower panel). To further validate these findings, we conducted a pull-down assay. The amount of GST-AtSET pulled down by His-AtCTD decreased when 1 × AtMTA was added, while the amount of GST-AtMTA pulled down by His-AtCTD was not affected by the addition of 1 × AtSET (Fig. 4B). This indicates that AtMTA can compete with AtSET for binding to the AtCTD of RNA Pol II. Moreover, as the concentration of GST-AtSET increased, the amount of GST-AtSET pulled down by His-AtCTD also increased, while the amount of GST-AtMTA pulled down decreased (Fig. 4B). We attempted to employ a truncated version of AtSET as a negative control. Initially, we divided AtSET into three segments (Additional file 1: Fig. S4A) and assessed their individual interactions with AtCTD and ATMTA. Our findings revealed that, excluding the AtSET3 and AtMTA group, all other group combinations exhibited interactions (Additional file 1: Fig. S4B and C). Subsequently, we conducted a pull-down assay by escalating the amount of GST, which served as a negative control (Additional file 1: Fig. S4D). Results indicated that the amount of GST-MTA pulled down by His-AtCTD remained unaffected by increasing GST (Additional file 1: Fig. S4D). These results collectively suggest that ATX1 and MTA compete for binding to the CTD of RNA Pol II.

### MTA competes ATX1 to interact with Ser5P-CTD of RNA Pol II in vitro and in vivo

**Fig. 5:**
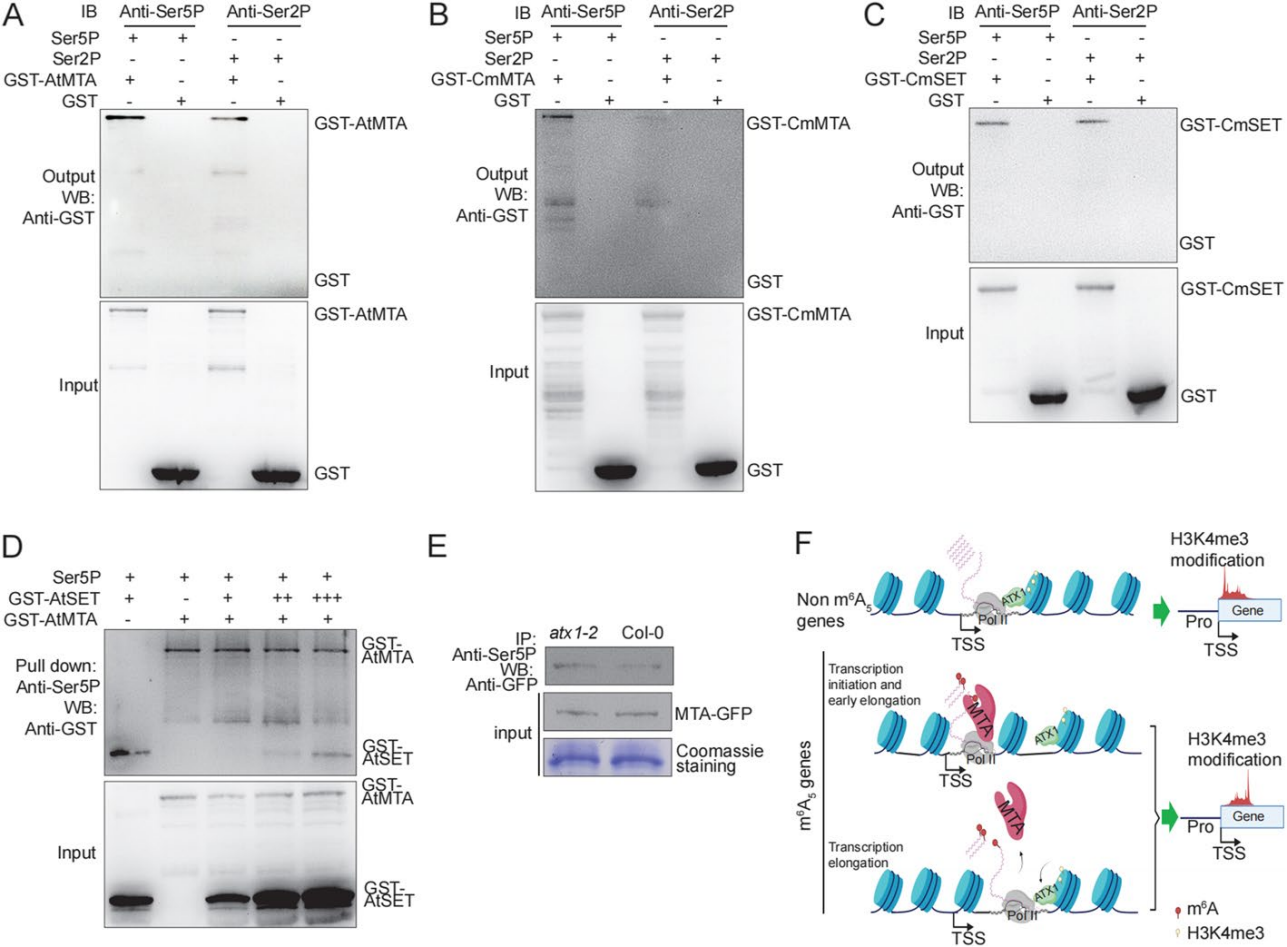
Binding of the SET or MTA to different phosphorylated forms of the CTD of Pol II. **A-C** Binding of AtMTA (**A**), CmMTA (**B**), CmSET (**C**) or GST to bead-bound peptides containing four consensus CTD heptad repeats (YSPTSPS) with phosphorylated at Ser5P or Ser2P were examined. The amount of GST or GST fused protein bound to the peptides on the beads was determined by immunoblot analysis with antibody to GST. **D** Pull down assay to examine the competition between AtMTA and AtSET for binding to Ser5P-CTD of RNA Pol II. Increasing concentrations of GST-AtSET was respectively incubated with a fixed concentration of GST-AtMTA. GST-fusion proteins were detected with an anti-GST antibody. Input means total protein lysate without immunoprecipitation. **E** Protoplast transient assay system to examine the competition between AtMTA and AtSET for binding to Ser5P-CTD of RNA Pol II. Protoplast cells obtained from *atx1-2* mutant and WT plants were used for *35S:AtMTA-GFP* transformation. Total proteins extracted from protoplast cells were immunoprecipitated with anti-Ser5P-CTD and analyzed by western blot with antibody to GFP. **F** Model for the m^6^A-mediated H3K4me3 shift on gene expression regulation. In cases where there is no m^6^A modification in the 5' UTR regions, histone methyltransferase ATX1 promotes H3K4me3 modification, leading to high level expression of genes. However, when the m^6^A writer MTA binds to the CTD domain of RNA Pol II, it competes with ATX1, resulting in reduced H3K4me3 modification during transcriptional initiation and early elongation stages. As the transcription elongation progresses and MTA dissociates from RNA Pol II, ATX1 is recruited by RNA Pol II to perform H3K4me3 modification on target genes. This downstream shift of H3K4me3 is associated with a decrease in gene expression levels

Phosphorylation of serine 5 (Ser5P) of the CTD is known to be a marker for transcription initiation and early elongation, while serine 2 (Ser2P) phosphorylation is associated with transcription elongation [39]. Previous studies have shown that the SET domain of AtATX1 preferentially binds to the Ser5P form of the CTD of RNA Pol II [37]. This led us to investigate whether MTA interacts with phosphorylated CTD. We performed pull-down assays using antibodies specific to Ser5P and Ser2P for capturing Ser5P-CTD and Ser2P-CTD, respectively. Our results revealed that AtMTA interacts with both Ser2P-CTD and Ser5P-CTD peptides, with a higher affinity observed for the Ser5P-CTD peptide (Fig. 5A). Similar findings were obtained with the MTA from chrysanthemum (Fig. 5B). However, CmSET exhibits equal interaction strength with both Ser2P-CTD and Ser5P-CTD (Fig. 5C).

To investigate whether ATX1 competes MTA for binding to Ser5P-CTD, we performed a pull-down assay using an anti-Ser5P antibody. Our findings revealed that GST-AtSET was predominantly outcompeted by GST-MTA (Fig. 5D, 1st lane *vs.* 3rd lane). Conversely, GST-MTA was only slightly competed by GST-SET (Fig. 5D, 2nd lane *vs.* 3rd lane), even though a higher amount of GST-SET was utilized in the pull-down assay (Fig. 5D, input). Additionally, as the concentration of GST-AtSET increased, the amount of GST-ATSET pulled down by anti-Ser5P exhibited a slight increase, while the amount of GST-AtMTA pulled down showed a slight decrease (Fig. 5D). These results indicate that AtMTA exhibits a higher binding affinity to the Ser5P-CTD of RNA Pol II compared to AtATX1.

To confirm these findings in an in vivo context, we generated GFP-fusion constructs of the AtMTA and introduced it into the protoplast derived from *atx1-2* mutant (Salk_149002C) [35] (Additional file 1: Fig. S3A) and WT plants. Compared to the cells without *AtATX1*, the amount of AtMTA-GFP co-immunoprecipitated by anti-Ser5P in cells with *AtATX1* (the WT cells) decreased (Fig. 5E), consistent with the results obtained from the pull-down assay. Collectively, these results suggest that during the transcription initiation and early elongation stage of m^6^A_5_ genes, Ser5P-CTD of RNA Pol II preferentially interacts with MTA for m^6^A synthesis, while ATX1 is displaced during this process, resulting in a downstream shift of H3K4me3 modification (Fig. 5F).

### ALKBH10B competes with MTA for binding to the Ser5P-CTD of RNA Pol II both in vitro and in vivo

**Fig. 6:**
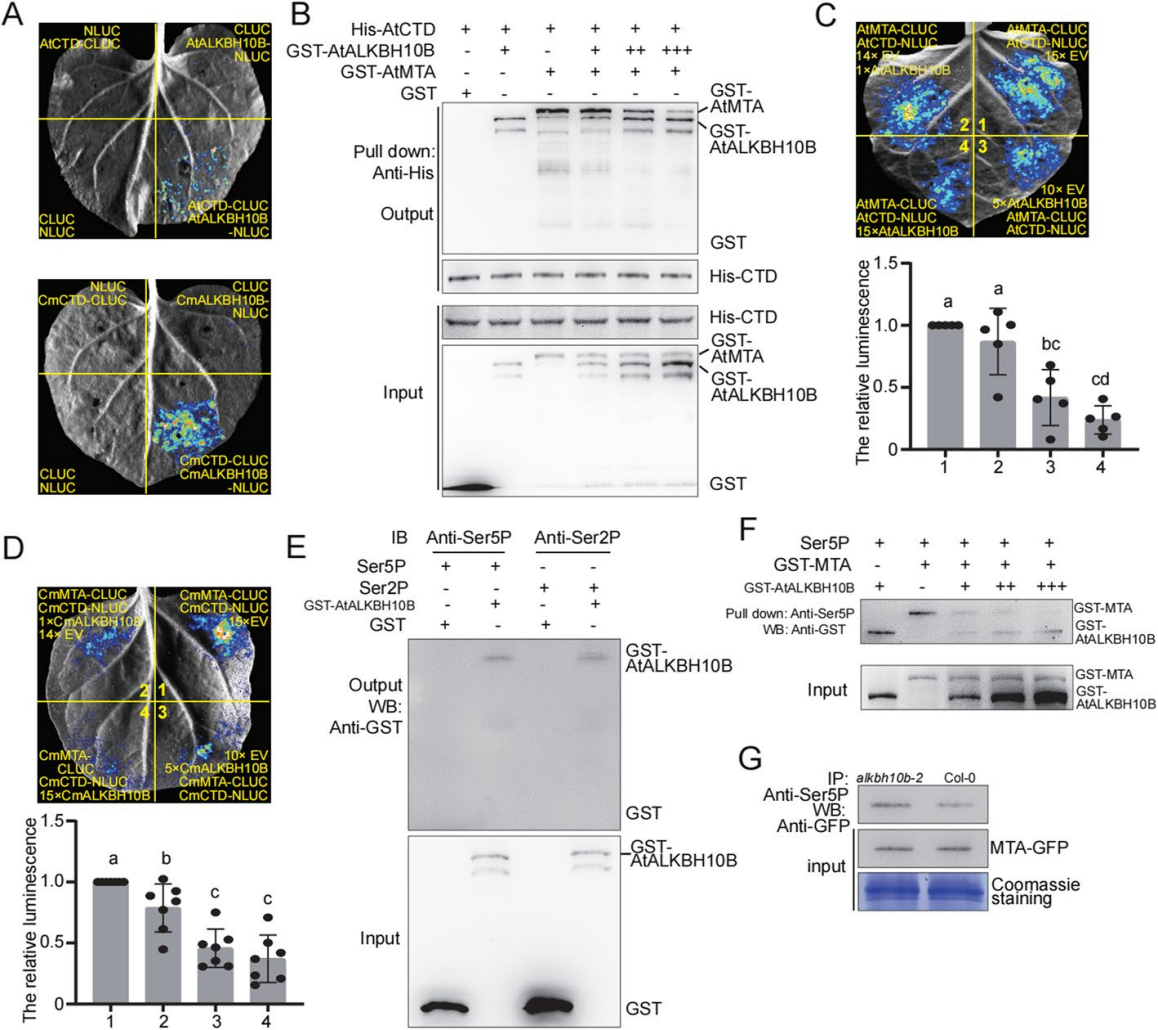
ALKBH10B competes with MTA for binding to the Ser5P-CTD of RNA Pol II. **A** Split-LUC assays examining the interaction between AtALKBH10B and AtCTD (upper panel), and CmALKBH10B and CmCTD (lower panel). Split-LUC assays were conducted by the infiltration of *Agrobacterium* carrying indicated constructs into *N. benthamiana* leaves. Images of plants sprayed with 500 μM luciferin and placed in the dark for 5 min are shown. **B** Pull-down assays to examine the interaction between AtALKBH10B or AtMTA and AtCTD. Increasing concentrations of GST-AtALKBH10B was respectively incubated with a fixed concentration of GST-AtMTA. **C-D** Split-LUC assays examining the competition between AtALKBH10B and AtSET (**C**), and CmALKBH10B and CmSET (**D**). Split-LUC assays were conducted by the infiltration of *Agrobacterium* carrying indicated constructs into *N. benthamiana* leaves. Images of plants sprayed with 500 μM luciferin and placed in the dark for 5 min are shown (upper panel). Quantitation of luciferase intensity from more than five biological replications are shown in plots (error bars are SD, lower panel). Different letters were used to indicate statistically significance difference (*P* < 0.05, Student’s t-test). **E** Binding of AtALKBH10B or GST to bead-bound peptides containing four consensus CTD heptad repeats (YSPTSPS) with phosphorylated at Ser5P or Ser2P were examined. The amount of GST or GST-AtALKBH10B protein bound to the peptides on the beads was determined by immunoblot analysis with antibody to GST. **F** Pull down assay to examine the competition between AtALKBH10B and AtMTA for binding to Ser5P-CTD of RNA Pol II. Increasing concentrations of GST-AtALKBH10B was respectively incubated with a fixed concentration of GST-AtMTA. **G** Protoplast transient assay system to examine the competition between AtMTA and AtALKBH10B for binding to Ser5P-CTD of RNA Pol II. Protoplast cells obtained from *alkbh10b-2* mutant and WT plants were used for 35S:AtMTA-GFP transformation. Total proteins extracted from protoplast cells were immunoprecipitated with anti-Ser5P-CTD and analyzed by western blot with antibody to GFP

Given the opposite effects of MTA and ALKBH10B on the shift of H3K4me3 (Fig. 3), we next investigated the role ALKBH10B in the MTA-CTD-ATX1 complex. Firstly, we performed Split-LUC assays, which revealed that ALKBH10B interacts with CTD of RNA Pol II in both *Arabidopsis* and chrysanthemum (Fig. 6A). Subsequently, pull-down assays were performed to confirm their interactions (Fig. 6B). Based on our observations, we hypothesized that ALKBH10B may compete with MTA for binding to RNA Pol II during transcription initiation. To test this hypothesis, split-LUC assays were conducted, and the results demonstrated that as the concentration of AtALKBH10B increased, the interaction between AtMTA and AtCTD significantly decreased in both *Arabidopsis* (Fig. 5C) and chrysanthemum (Fig. 5D). Furthermore, the pull down-assays indicated that with an increase in the concentration of GST-AtALKBH10B, the amount of GST-AtALKBH10B pulled down by His-AtCTD also increased, while the amount of GST-AtMTA pulled down decreased (Fig. 6B). Collectively, these results strongly suggest a competitive binding relationship between ALKBH10B and MTA for the CTD of RNA Pol II.

We then examined whether the competition happened on the Ser5P-CTD of RNA Pol II. Pull down assays were conducted, revealing that AtALKBH10B interacts with both Ser2P-CTD and Ser5P-CTD peptides (Fig. 6E). Furthermore, we observed that GST-AtMTA was competitively displaced by GST-ALKBH10B when pulled down by Ser5P-CTD (Fig. 6F). As the concentration of GST-AtALKBH10B increased, the amount of GST-ATALKBH10B pulled down by anti-Ser5P exhibited a slight increase, while the amount of GST-AtMTA pulled down decreased (Fig. 6F). These results indicate that ALKBH10B could compete MTA at Ser5P-CTD of RNA Pol II. To further support this, a protoplast transient assay was performed, demonstrating that the amount of AtMTA-GFP co-immunoprecipitated by anti-Ser5P in cells with *AtALKBH10B* (Col-0) decreased compared to cells without *AtALKBH10B* (Fig. 6G), consistent with the results obtained from the pull-down assay. Collectively, these results strongly suggest that ALKBH10B plays a role in determining the occupancy of MTA at transcriptional initiation.

### m^6^A-mediated H3K4me3 shift in the 5’ UTR is involved in leaf senescence

**Fig. 7:**
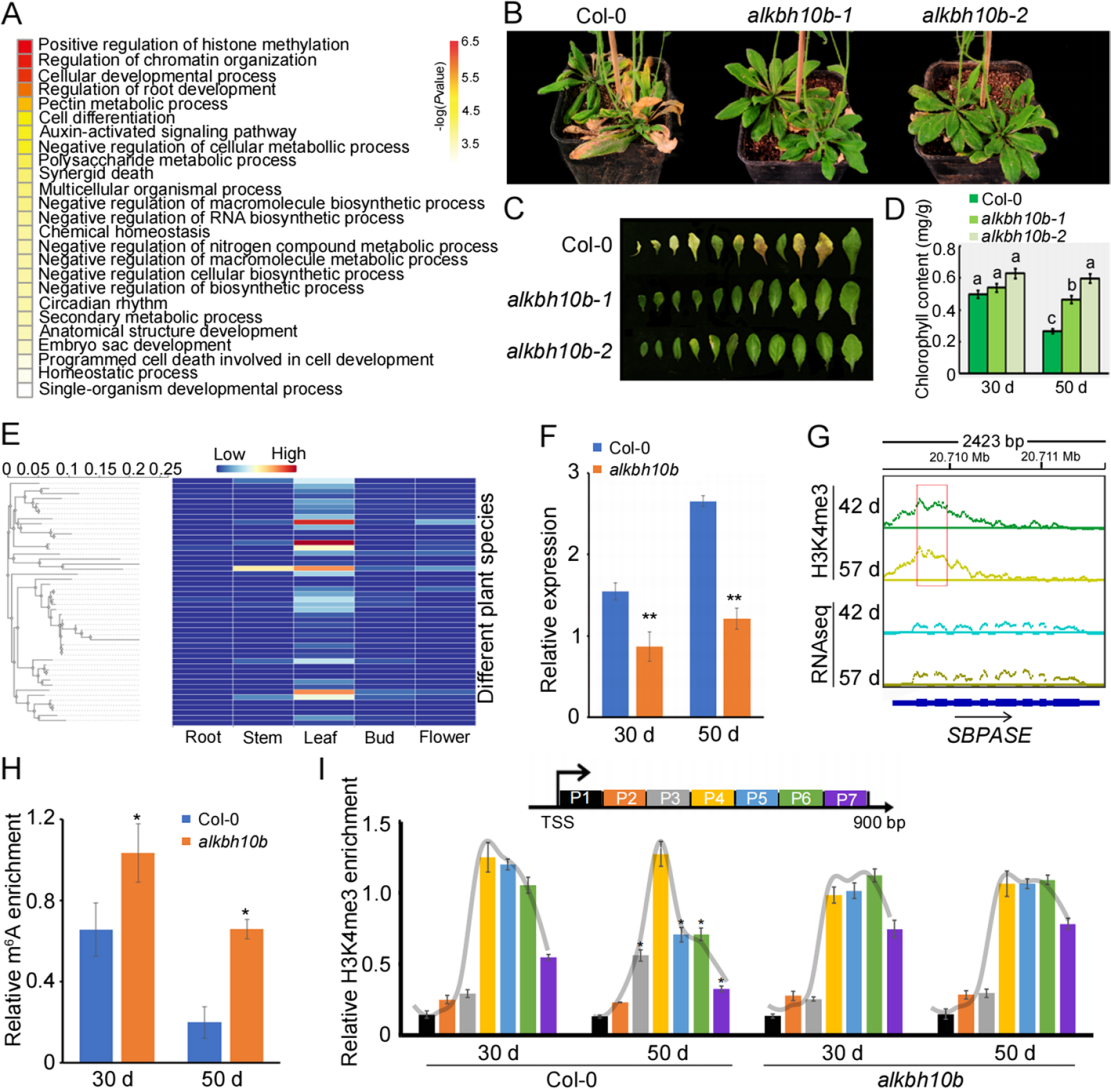
m^6^A-mediated H3K4me3 shift in the 5’ UTR is involved in leaf senescence. **A** Gene ontology (GO) biological processes enrichment analyses of genes with m^6^A modification only in 5’ UTR regions. **B** The senescence phenotype of 50 d old Col-0, *alkbh10b-1* and *alkbh10b-2* plants. **C** The senescence phenotype of leaves. The leaves were detached and arranged according to their age. **D** Total chlorophyll contents in leaves from Col-0, *alkbh10b-1* and *alkbh10b-2* plants were measured. Three biological replicates were performed. Error bars represent SD. Different letters indicate a statistically significant difference with *P* ≤ 0.05 by Student's t-test. **E** The expression analysis of *SBPASE* from different plant species was performed using GERDH database (https://dphdatabase.com/). Left panel showing the phylogenetic tree of SPBASE protein from different plant species; right panel showing the expression of *SPBASE* gene in root, stem, leaf, bud and flower across various plant species. **F** Quantitative PCR detection of *SBPASE* expression in Col-0 and *alkbh10b-2* plants at 30 d and 50 d. ** indicates *p* value < 0.01 by Student's t-test. **G** Genome browser traces of H3K4me3 ChIP-seq data and RNA-seq data from *SBPASE* gene during leaf senescence in *Arabidopsis*. Red box highlights the different enrichment of H3K4me3. **H** m^6^A RNA immunoprecipitation quantitative PCR (m^6^A-IP-qPCR) detection of m^6^A modification enrichment on *SBPASE* gene in Col-0 and *alkbh10b-2* plants at 30 d and 50 d. Total RNA was fragmented and immunoprecipitated with anti-m^6^A antibody. Both input control and m^6^A-IP samples were subjected to qPCR analysis. * indicates *p* value < 0.05 by Student's t-test. **I** ChIP-qPCR detection of H3K4me3 enrichment on *SBPASE* gene in Col-0 and *alkbh10b-2* plants at 30 d and 50 d. The sonicated chromatin was immunoprecipitated using anti-H3K4me3 antibody, DNA was eluted and amplified by primers (P1-P7) indicated in the figure. Trendlines were added to trace the H3K4me3 shift. * indicates *p* value < 0.05 by Student's t-test between 30 and 50 days for each primer in Col-0 or *alkbh10b* plants

To gain deeper insights into the function of the m^6^A_5_ genes, we performed a Gene Ontology (GO) analysis and found their enrichment in regulation of histone methylation, development, cell death, and metabolic processes (Fig. 7A). Previous studies have demonstrated that *alkbh10b-1* (SALK_004215C; Additional file 1: Fig. S5) and *alkbh10b-2* (SALK_107289C) mutants exhibit delayed flowering and suppressed vegetative growth [12]. Additionally, both mutants display a delayed leaf senescence phenotype (Fig. 7B-D). To unravel the mechanisms underlying m^6^A-mediated leaf senescence, we re-evaluated the m^6^A_5_ genes and observed that the *SEDOHEPTULOSE-BISPHOSPHATASE* (*SBPASE*) gene is predominantly expressed in leaf tissues across various plant species (Fig. 7E). Moreover, mutations in the *SBPASE* gene result in a notable delay in developmental leaf senescence and flowering time [40], similar to the phenotype observed in *alkbh10b* mutants (Fig. 7B-D). Notably, we observed lower expression levels of *SBPASE* in *alkbh10b* plants compared to WT plants (Fig. 7F). Furthermore, we re-analyzed the H3K4me3 ChIPseq data (GSE67776) obtained from green leaves at 42 days and senescent leaves at 57 days [41]. Our analysis revealed an upstream shift in H3K4me3 during leaf senescence that resulted in increased *SBPASE* expression (Fig. 7G), which is consistent with our qRT-PCR results using green and senescent leaves harvested at 30 and 50 days, respectively (Fig. 7F). To confirm this regulatory mechanism, we performed m^6^A-qPCR on green and senescent leaves of both Col-0 and *alkbh10b* plants. The results showed down-regulation of m^6^A modification on the 5’UTR region of *SBPASE* during senescence in both Col-0 and *alkbh10b*, with *alkbh10b* exhibiting higher levels of m^6^A modification (Fig. 7H). Furthermore, we examined H3K4me3 modification downstream of the TSS region of *SBPASE* and observed an upstream shift during senescence, whereas no significant shift was observed in *alkbh10b* (Fig. 7I). These findings collectively indicate that *SBPASE* regulates leaf senescence controlled by m^6^A-mediated H3K4me3 shift in 5’ UTR.

In summary, all these results suggested that the deposition of m^6^A in the 5’ UTR alters H3K4me3 modification, finely tuning the expression of target genes and contributing to leaf senescence.

## Discussion

Previous studies have primarily focused on m^6^A modifications in the vicinity of stop codons and 3’ untranslated regions (UTRs), rather than the 5’ UTRs [25, 42]. The molecular functions of 3’ UTR-specific m^6^A modifications have been extensively investigated. They have been found to be associated with alternative polyadenylation of transcripts [43, 44], and the regulation of gene expression [45]. In spermatocytes and round spermatids, proper erasure of m^6^A in the 3’ UTR is crucial for correct splicing and stability of mRNA molecules with long 3’ UTR [46]. On the other hand, 5’ UTR-specific m^6^A modifications have received less attention but have been shown to play essential roles in regulating cortical development [13], and cellular stress responses [14, 15] at post-transcriptional level. However, the roles of 5’ UTR-specific m^6^A modifications in transcriptional regulation have received limited attention. In this study, we found that m^6^A methylation deposited in the 5’ UTR regions potentially contributes to gene repression, and this phenomenon is conserved in *Arabidopsis*, rice and chrysanthemum (Fig. 1). Furthermore, we made a notable discovery that gene expression is regulated by a downstream shift of H3K4me3, controlled by m^6^A modification in the 5’ UTR regions, which is implicated in the regulation of leaf senescence. Nevertheless, we cannot overlook the potential impact of m^6^A on mRNA stability, translational efficiency, alternative splicing, and other factors, leading to varied expression dynamics of genes/proteins beyond m^6^A_5_, thereby influencing leaf senescence. Furthermore, the precise functional significance of the H3K4me3 shift remains to be fully elucidated. Additional evidence is necessary to address the unanswered questions concerning the functions and underlying mechanisms of H3K4me3 shift in the regulation of gene expression in future studies.

To date, there have been limited studies on the shift of histone modification in genes with m^6^A methylation. One study reported that when the transcriptional status of a gene changes from silenced to activated, the first nucleosome downstream of TSS shifts in the downstream direction [47], indicating that nucleosome positioning plays an important role in gene expression regulation. In addition, a recent report showed that incorporation of N1-methylpseudouridylation into mRNA causes + 1 ribosomal frameshifting in vitro and in vivo [48], indicating the effect of mRNA modification in translational regulation. In our study, we observed a shift in H3K4me3, mostly less than 150 bp, which is not sufficient to bypass a nucleosome, suggesting a repositioning of the nucleosome.

Furthermore, it has been reported that the m^6^A writer protein METTL14 interacts with H3K36me3 on chromatin and guides N6-methylation on nascent RNAs, leading to a higher level of N6-methylation in long exons [20]. A recent study demonstrated that super-enhancer RNA m^6^A (seRNA m^6^A) promotes local chromatin accessibility and facilitates the transcription of oncogenes. This effect is mediated by the seRNA m^6^A-YTHDC2 module, which recruits the H3K4 methyltransferase MLL1 to promote H3K4me3 modification [19]. These findings suggest a connection between mRNA m^6^A modification and histone modifications. Profiling of histone modifications upon m^6^A loss via *METTL14* knockout revealed increased levels of H3K4me3, H3K27me3 and H3K27ac in cell-proliferation-related genes [49]. This finding can be interpreted in light of our results, which indicate that MTA and ATX1 compete to interact with RNA Pol II. Loss of H3K4me3 has been reported to have minimal effects on transcriptional initiation but leads to a widespread decrease in transcriptional output, an increase in RNA Pol II pausing and slower elongation [50]. In our study, we found that MTA binding to the CTD domain of RNA Pol II competes with ATX1, resulting in lower H3K4me3 modification at the transcription initiation and early elongation process. When MTA dissociates from RNA Pol II during transcription elongation, RNA Pol II recruits ATX1 for H3K4me3 modification of target genes, leading to a downstream shift of H3K4me3 modification and lower level of gene expression (Fig. 5F). The MTA binding to RNA Pol II results in H3K4me3 loss, which may lead to increased RNA Pol II pausing and slower early elongation, consequently resulting in decreased transcriptional output. In addition, it is important to consider the possibility that m^6^A modification may impact mRNA stability, leading to reduced transcription levels. Furthermore, investigating whether the enzymatic activity of MTA or ALKBH10B is necessary for the regulation of gene expression through H3K4me3 shift would be an intriguing aspect to explore in future studies.

## Conclusions

In conclusion, our findings indicate that m^6^A-mediated shifts in H3K4me3 contributing to gene repression and leaf senescence. These findings provide valuable insights into the mechanism underlying m^6^A-mediated gene expression regulation in the 5' UTR regions, opening up promising avenues for further research in this field.

## Methods

### Plant materials

The cut flower chrysanthemum cultivar, ‘Hualing’, was obtained from Chrysanthemum Germplasm Resource Conservation Center in Nanjing Agricultural University (Nanjing, China). The *alkbh10b-1* (Salk_004215C) *Arabidopsis* seeds were kindly donated by Dr. Jungnam Cho, and the *atx1-2* (Salk_149002C) and *alkbh10b-2* (Salk_107289C) mutant lines were obtained from *AraShare* (www.arashare.cn).

### Assay for natural and dark-induced leaf senescence

To investigate age-dependent leaf senescence, we selected the third and fourth rosette leaves of individual *Arabidopsis* plants. These leaves were used for various analyses, including chlorophyll content measurement, gene expression analysis, and examination of m^6^A and H3K4me3 modifications.

For the induction of dark-induced leaf senescence, the selected leaves were excised and placed in petri dishes containing wet paper. The petri dishes were wrapped with double-layer aluminum foil and incubated at a temperature of 22 °C throughout the experiment.

### Measurements of chlorophyll content

The detached leaves were incubated in 80% acetone (v/v) in darkness for 24 h. Then the absorbance of the solution was measured at 645 and 657 nm using a spectrophotometer. The chlorophyll content was calculated using the formula: (20.2 × A_645_ + 8.02 × A_657_)/g fresh weight.

### m^6^A-immunoprecipitation and sequencing (m^6^A-seq) and analysis

mRNAs were fragmented into 100-nucleotide fragments and incubated with 0.5 mg/ml anti-m^6^A polyclonal antibody (202–003; Synaptic Systems). Bound mRNA was eluted and used to construct the libraries. Sequencing was performed on the Illumina Hiseq 2500 platform.

Clean reads were aligned to reference genomes using bowtie2 [51]. The peak-caller MACS2 [52] was used for peak detection with FDR value was less than 0.01 and fold enrichment more than 1.5.

### RNA-seq processing and analysis

Total RNA was used to prepare RNA-seq libraries using TruSeq RNA Library Prep Kit (Illumina). Multiplexed libraries were sequenced on an Illumina HiSeq 2500. Clean reads were aligned to the reference genome using HISAT2 [53]. DEGs were identified using Cufflinks [54], and detected with |log2(fold change)| and RPKM values larger than 1. The expression analysis of *SBPASE* was performed using GERDH database [55].

### ChIP-seq processing and analysis

Chromatin immunoprecipitation was performed according to published protocol [56]. Clean reads were mapped to the reference genome using bowtie2 [51]. Peaks significantly enriched in ChIP-seq tags were identified using MACS2 [52].

Chromatin-immunoprecipitated DNA was amplified by primers corresponding to genes of interest. Primers used for ChIP-qPCR are listed in Additional file 2: Table S2.

### Western blot

To analyze the proteins, we performed SDS–PAGE followed by electroblotted onto a nitrocellulose membrane. The membrane was probed with specific primary antibodies and then secondary antibodies. The signals were visualized using the SuperSignal West Pico PLUS kit (ThermoFisher).

### Pull-down assay

The fusion proteins were incubated together and subsequently washed using a pull-down buffer. The precipitated magnetic beads were collected and then resuspended in a protein extraction buffer. The proteins were then separated by SDS-PAGE and detected using specific antibodies.

### Immunoprecipitation assays

The harvested cells were incubated in co-IP buffer on ice for 30 min, allowing the proteins to be solubilized in the co-IP buffer. After following centrifugation, the cleared extract was then combined with anti-Ser5P-CTD antibody and incubated overnight at 4 °C. The magnetic beads were collected and resuspended in a protein extraction buffer. The proteins were then separated by SDS-PAGE and detected using a specific antibody.

### *N. benthamiana* transient expression assay

The Agrobacterium strains were injected into *N. benthamiana* plants. 2 days later, the leaves of the plants were sprayed with 500 μM luciferin (Promega) and examined using a CCD imaging apparatus (Tanon, Shanghai, China).

## Supplementary Information

Additional file 1: Fig. S1. m^6^A methylation in *Arabidopsis* and *Oryza sativa*. Fig. S2. m^6^A methylation deposits in the 5’ UTR of mRNA shifted H3K4me3 histone modification. Fig. S3. Genotyping of T-DNA insertion mutants and m^6^A levels in different plants. Fig. S4. The negative control of MTA and ATX1 interact with CTD of RNA Pol II. Fig. S5. The diagram to show the T-DNA insertion for *alkbh10b-1*

Additional file 2: Table S1. Summary of reads and mapped rates in different samples. Table S2. Primers used in this paper

Additional file 3: Uncropped images of Western blots in Fig. 4, Fig. 5, Fig. 6 and Fig. S4

Additional file 4: Review history.

## Data Availability

All data supporting the findings of this study are available in the article and its supplementary figures and tables. The raw meRIP-, ChIP- and RNA-sequencing data reported in this paper are available at the Genome Sequence Archive in National Genomics Data Center, Chinese Academy of Sciences, with accession number CRA009857 [57].
