# Supplementary material for: mRNA m6A regulates gene expression via H3K4me3 shift in 5’ UTR
Source: Genome Biol. 2025 Mar 12;26:54. doi: 10.1186/s13059-025-03515-8 (PMC11900566; doi:10.1186/s13059-025-03515-8)
Supplement: Supplementary file 1 — Additional file 1: Fig. S1. m6A methylation in Arabidopsis and Oryza sativa. Fig. S2. m6A methylation deposits in the 5’ UTR of mRNA shifted H3K4me3 histone modification. Fig. S3. Genotyping of T-DNA insertion mutants and m6A levels in different plants. Fig. S4. The negative control of MTA and ATX1 interact with CTD of RNA Pol II. Fig. S5. The diagram to show the T-DNA insertion for alkbh10b-1 [file 13059_2025_3515_MOESM1_ESM.pdf]

Additional File 1

1. Fig. S1. m^6A methylation in *Arabidopsis* and *Oryza sativa*.

2. Fig. S2. m^6A methylation deposits in the 5' UTR of mRNA shifted H3K4me3 histone modification.

3. Fig. S3. Genotyping of T-DNA insertion mutants and m^6A levels in different plants.

4. Fig. S4. The negative control of MTA and ATX1 interact with CTD of RNA Pol II.

5. Fig. S5. The diagram to show the T-DNA insertion for *alkbh10b-1*.

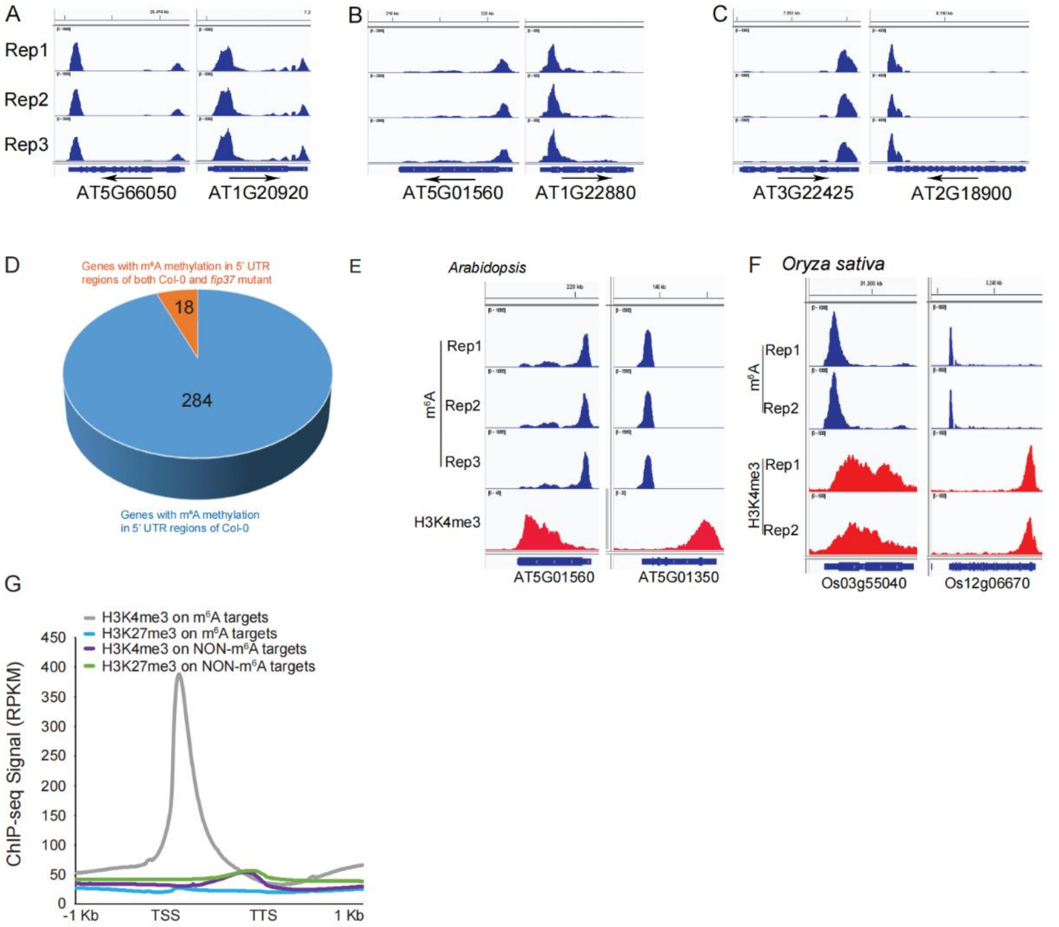

Fig. S1. m^{6}A methylation in *Arabidopsis* and *Oryza sativa*. **A-C** Genes with m^{6}A methylation in both 5' UTR and 3' UTR region (m^{6}A$_{53}$) (**A**), genes with m^{6}A methylation only in 5' UTR region (m^{6}A$_5$) (**B**) and genes with m^{6}A methylation only in 3' UTR region (m^{6}A$_3$) (**C**) were shown. **D** The genes with m^{6}A modification in 5' UTR regions uniquely detected in Col-0 (blue), and shared with *fip37* mutant (orange). **E** Genome browser traces of m^{6}A-seq and H3K4me3 ChIP-seq data from representative genes in *Arabidopsis*. **F** Genome browser traces of m^{6}A-seq and H3K4me3 ChIP-seq data from representative genes in *Oryza sativa*. **G** Distribution of H3K4me3 or H3K27me3 reads in transcript segments with or without m^{6}A modification.

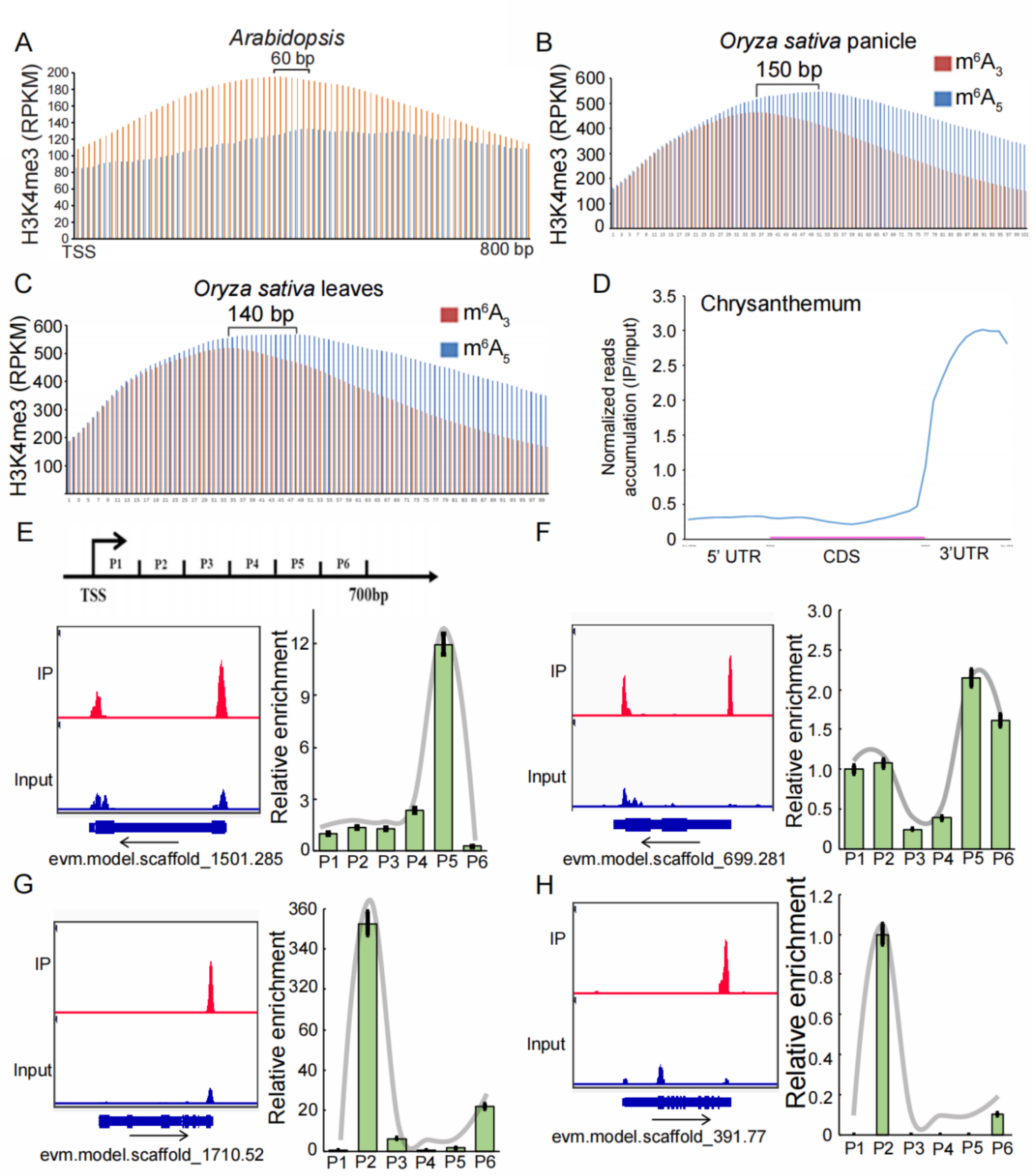

Fig. S2. m^{6}A methylation deposits in the 5' UTR of mRNA shifted H3K4me3 histone modification. **A-C** Bar plots show the normalized H3K4me3 signal downstream of TSS in leaves of *Arabidopsis* (**A**), panicle (**B**) and leaves (**C**) of *Oryza sativa*. Normalized H3K4me3 signal downstream of the TSS were binned in 10-bp intervals, and the distribution was plotted. The distance between summits was indicated in the figure. m^{6}A$_3$: genes with m^{6}A methylation only in 3' UTR region; m^{6}A$_5$: genes with m^{6}A methylation only in 5' UTR region. **D** Accumulation of m^{6}A-IP reads along transcripts in chrysanthemum. Each transcript was divided into three parts: 5' UTRs, CDS and 3' UTRs. **E-H** Genome browser traces and ChIP-qPCR to show the m^{6}A enrichment and H3K4me3 modification, respectively, for genes with m^{6}A modification specifically in 5' UTR regions (**E-F**) and 3' UTR regions (**G-H**). About 700 bp regions were divided into 6 parts for ChIP-qPCR examination.

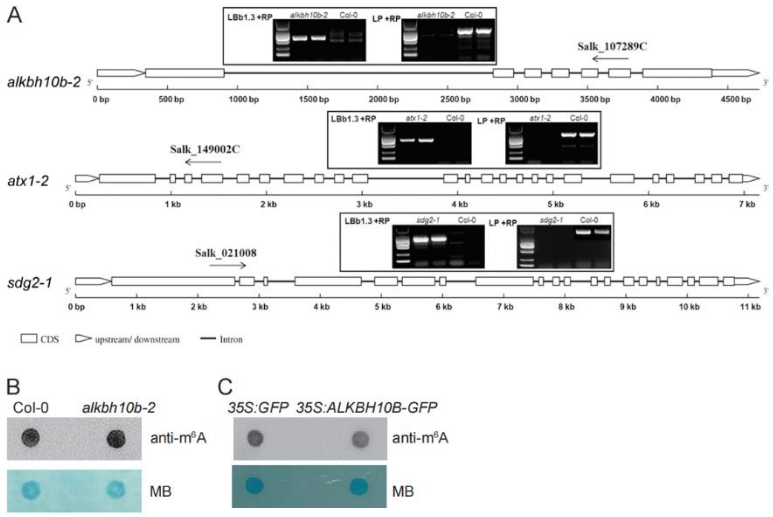

Fig. S3. Genotyping of T-DNA insertion mutants and m^{6}A levels in different plants. **A** The diagram to show the T-DNA insertion for *alkbh10b-2*, *atx1-2* and *sdg2-1* mutants. Genotyping results were shown. **B-C** m^{6}A dot-blot assay to illustrate the overall level variances between Col-0 and *alkbh10b-2* mutant (**B**), and the global change in m^{6}A modification in cells expressing *35S:AtALKBH10B-GFP* (**C**).

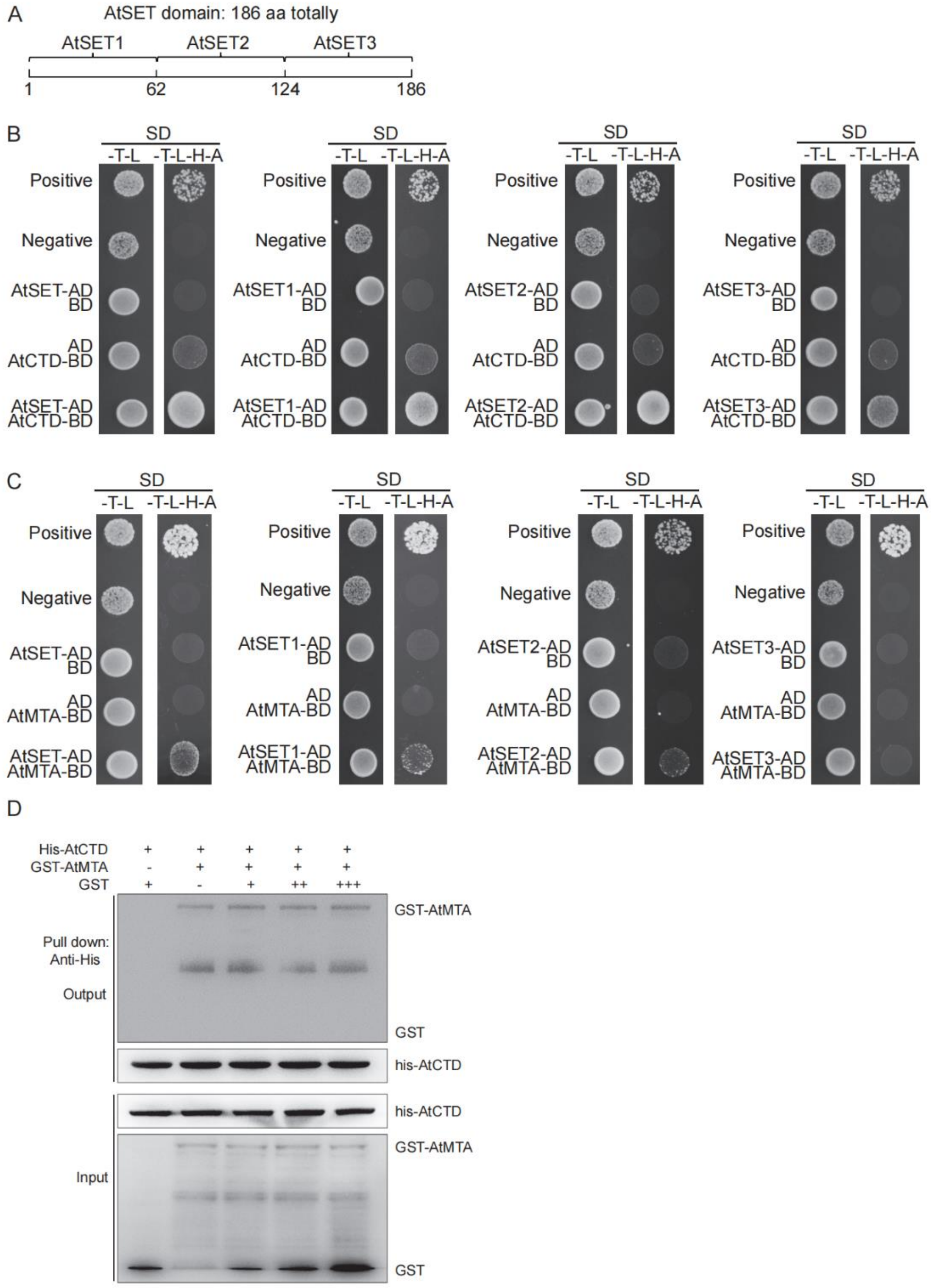

Fig. S4. The negative control of MTA and ATX1 interact with CTD of RNA Pol II. **A** The diagram to show the 3 fragments of AtSET domain (AtSET1-3) used for the following assays. **B-C** Y2H assay indicates the interaction between AtCTD and AtSET fragments (**B**), as well as between AtMTA and AtSET fragments (**C**). **D** Pull-down assays to examine the interaction between GST or AtMTA and AtCTD. Increasing concentrations of GST was respectively incubated with a fixed concentration of GST-AtMTA. GST-fusion proteins and GST were detected with an anti-GST antibody. Input means total protein lysate without immunoprecipitation.

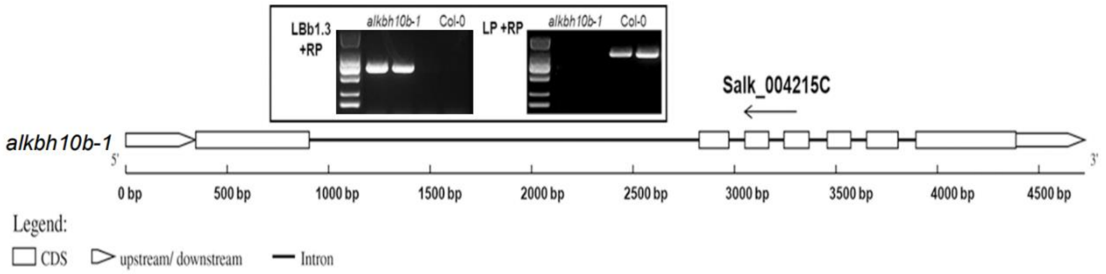

Fig. S5. The diagram to show the T-DNA insertion for *alkbh10b-1*. Genotyping results were shown.
