# Supplementary material for: mRNA m6A regulates gene expression via H3K4me3 shift in 5’ UTR
Source: Genome Biol. 2025 Mar 12;26:54. doi: 10.1186/s13059-025-03515-8 (PMC11900566; doi:10.1186/s13059-025-03515-8)
Supplement: Supplementary file 2 — Additional file 2: Table S1. Summary of reads and mapped rates in different samples. Table S2. Primers used in this paper [file 13059_2025_3515_MOESM2_ESM.pdf]

Additional File 2

Table S1. Summary of reads and mapped rates in different samples.

Libraries	total clean reads	mapped reads	mapped ratio
atx1-IP_meRIP-seq	19510348	14267917	73.13%
atx1-IN_meRIP-seq	20627757	13300778	64.48%
sdg2-IP_meRIP-seq	20654387	14619175	70.78%
sdg2-IN_meRIP-seq	20009030	12911827	64.53%
Chrysanthemum-IP_meRIP-seq	21941942	15570002	70.96%
Chrysanthemum-IN_meRIP-seq	23951865	12871732	53.74%
alkbh10b_H3K4me3	14859333	9377179	63.11%
Chrysanthemum_RNA-seq_rep1	49950277	44046154	88.18%
Chrysanthemum_RNA-seq_rep2	49693447	42527652	85.58%

Table S2. Primers used in this paper.

Primer name	sequence	Purpose
1751.54-P1-F	AAAACCAATGTACCGTGTTTATGA	ChIP-qPCR
1751.54-P1-R	TCGGAAAAGCAACTCTAATCG	ChIP-qPCR
1751.54-P2-F	GAAACCTCACAACCGCAGAT	ChIP-qPCR
1751.54-P2-R	GAAGTTGCATATTTCTTTCCGTTT	ChIP-qPCR
1751.54-P3-F	TCCATCAACACATTGTTCACAC	ChIP-qPCR
1751.54-P3-R	CCATTCTTTGCGCCATAAGT	ChIP-qPCR
1751.54-P4-F	CGCATTTCAAACCAATGTCTT	ChIP-qPCR
1751.54-P4-R	CAGTCCTCACATCATAGTATTCATCA	ChIP-qPCR
1751.54-P5-F	AGGATTGCCATATCGAAGCA	ChIP-qPCR
1751.54-P5-R	TGCAACAGAACAAACAATTACCA	ChIP-qPCR
1751.54-P6-F	TGCCGGCACAAAGAATCTA	ChIP-qPCR
1751.54-P6-R	TGCAACCGTATCTGCTTACTTT	ChIP-qPCR
699.281-P1-F	TGTAAAACTGTTGTATTTGAGGTTG	ChIP-qPCR
699.281-P1-R	GCGGGAGTGCTTTGTCAATA	ChIP-qPCR
699.281-P2-F	GCTACCCCGCTCGTTAATAG	ChIP-qPCR
699.281-P2-R	TCCGTCTAGGAGACGAATGA	ChIP-qPCR
699.281-P3-F	TTTTTCTGCTCAAATAATGTGC	ChIP-qPCR
699.281-P3-R	TTGATGAGAATCCCTTCGATTT	ChIP-qPCR
699.281-P4-F	AAATCGAAGGGATTCTCATCA	ChIP-qPCR
699.281-P4-R	AATAGCAAAACGATGATTGAATTT	ChIP-qPCR
699.281-P5-F	TGATCTTAAATTACATCGATTCTGA	ChIP-qPCR
699.281-P5-R	GACCCACGAGCCACTTTTT	ChIP-qPCR
699.281-P6-F	GTGGGTCAAGGCACAGTGTT	ChIP-qPCR
699.281-P6-R	ACACACACGCACACACACAC	ChIP-qPCR
1501.285-P1-F	CAGCCGATAGAAAAGGAGAAGA	ChIP-qPCR

1501.285-P1-R	TTGTTGTTGTTGTTGGAGCA	ChIP-qPCR
1501.285-P2-F	CAAAACCAACAATCCCAACA	ChIP-qPCR
1501.285-P2-R	GTTGGAAGGGAAGCGAGAG	ChIP-qPCR
1501.285-P3-F	ACAACAACCTCGTTCCCAAC	ChIP-qPCR
1501.285-P3-R	TCGGAGTGGGTCTAGTTTGC	ChIP-qPCR
1501.285-P4-F	GGGTGGTGTTGGGTCTCC	ChIP-qPCR
1501.285-P4-R	CTGGCAAAGGGCTTCTTAAA	ChIP-qPCR
1501.285-P5-F	CCAGAGGTTTGTCCGACTGT	ChIP-qPCR
1501.285-P5-R	GTTTGTCCGCACGCTTCA	ChIP-qPCR
1501.285-P6-F	GGTTATTTAGGGGTTACGAAGC	ChIP-qPCR
1501.285-P6-R	CATAAGCCAACAGTCGACCA	ChIP-qPCR
1710.52-P1-F	CGAAAACCAAAATCCTTTCTAA	ChIP-qPCR
1710.52-P1-R	TGGTTTTCTTGGGGTTTTTG	ChIP-qPCR
1710.52-P2-F	AAACCCCAAGAAAACCATCA	ChIP-qPCR
1710.52-P2-R	TCTTTTGGAGGTGGGTTTTG	ChIP-qPCR
1710.52-P3-F	GGGTTTCATCAGACGATGTTC	ChIP-qPCR
1710.52-P3-R	CCACCATTGCAACAACCTTA	ChIP-qPCR
1710.52-P4-F	TTGGCTTTGTGTAACGCTGA	ChIP-qPCR
1710.52-P4-R	ACCAGCAAAAGACTGACTCCA	ChIP-qPCR
1710.52-P5-F	TGTTATATAGGATATAGGTTAGAATGTTGG	ChIP-qPCR
1710.52-P5-R	GGGGGCATTACACTACCACT	ChIP-qPCR
1710.52-P6-F	GTCGGGTATCCTTCCAGGTT	ChIP-qPCR
1710.52-P6-R	AATACTTTCTAAGAATCAGACCTGACA	ChIP-qPCR
811.85-P1-F	AAAAGAAAAAGAAAAAGAAAAAGATGG	ChIP-qPCR
811.85-P1-R	CTATCTATCACCGTCAATCTATCTATCTA	ChIP-qPCR
811.85-P2-F	TCTTCACTTTATTTGTTAACCGTTATTT	ChIP-qPCR
811.85-P2-R	GCGCCATTAACGGAAAAAG	ChIP-qPCR
811.85-P3-F	CCCCACCCCACACTAACATA	ChIP-qPCR

811.85-P3-R	TCAGCTGCTAATTCAATTCTTCTT	ChIP-qPCR
811.85-P4-F	GGTGCAAATAAATAAATCTGGTGA	ChIP-qPCR
811.85-P4-R	TCCAGACATCACCAAAACATC	ChIP-qPCR
811.85-P5-F	GATGTTTTGGTGATGTCTGGA	ChIP-qPCR
811.85-P5-R	CAATAACTAAATTAACATCCAATTAATCA	ChIP-qPCR
811.85-P6-F	GCTAATTAAGTTTAGTAATTTAGTTTACTA	ChIP-qPCR
811.85-P6-R	GGAGTAATAACACAATTAGTCAATTAACC	ChIP-qPCR
391.77-P1-F	CATCACACCACACACATTTCTCT	ChIP-qPCR
391.77-P1-R	TGATTTGATTTGGGGTAAAATG	ChIP-qPCR
391.77-P2-F	TTGCAACAACTGAAACTGGAA	ChIP-qPCR
391.77-P2-R	TGTTGTAATTAAGCTTCTGAGTTATGAA	ChIP-qPCR
391.77-P3-F	AAGAAGAGGTAAAACAAGAAGATTTG	ChIP-qPCR
391.77-P3-R	GGCATCATTTTCAAGAATCCA	ChIP-qPCR
391.77-P4-F	TTGCTAATTGTACTTTGATGCAGA	ChIP-qPCR
391.77-P4-R	GCAAAAATTATTTGGAATTGCTT	ChIP-qPCR
391.77-P5-F	GATTTGGGACTTGGTCGAAA	ChIP-qPCR
391.77-P5-R	TTTCCATGCTAGGGAATAGTGAA	ChIP-qPCR
391.77-P6-F	CATGACTAAAGCGGTAGTTAATTTTT	ChIP-qPCR
391.77-P6-R	CGAAGTCAATCTTGACGGAAA	ChIP-qPCR
391.77-P3-F	AAGAAGAGGTAAAACAAGAAGATTTG	ChIP-qPCR
391.77-P3-R	GGCATCATTTTCAAGAATCCA	ChIP-qPCR
391.77-P4-F	TTGCTAATTGTACTTTGATGCAGA	ChIP-qPCR
SBPASE-CHIP-1F	GTTAACTTCTACTTGCATAGCCACAC	ChIP-qPCR
SBPASE-CHIP-1R	TGGTTATGAGATTCTATTATCTTCAATTTC	ChIP-qPCR
SBPASE-CHIP-2F	AAAGCCTCTCAACACATCTCTTTG	ChIP-qPCR
SBPASE-CHIP-	ACACGTGGCAGTAACTGAAGAAGATA	ChIP-qPCR

2R		
SBPASE-CHIP-3F	GTCCACTTGTGTTCTTGATTATTTTG	ChIP-qPCR
SBPASE-CHIP-3R	ATGCTGGTCTCCATTGTTAGAAG	ChIP-qPCR
SBPASE-CHIP-4F	GTCTCTTCTCAACGATCCTCTACATT	ChIP-qPCR
SBPASE-CHIP-4R	AACAAATCCTACACTGAAGATAAGCA	ChIP-qPCR
SBPASE-CHIP-5F	AATTTGATAAAAGCAGCGTCTAAAAT	ChIP-qPCR
SBPASE-CHIP-5R	CTCTTAGCTTTTGTGGCTTTAAGTTG	ChIP-qPCR
SBPASE-CHIP-6F	AAGCTTGGTTGAGTAACACAAAGAC	ChIP-qPCR
SBPASE-CHIP-6R	CATTCAAAAGTAAGAAACTTTAACTGAA	ChIP-qPCR
SBPASE-CHIP-7F	GATTGAGAACTTTGCTGATGTGTATG	ChIP-qPCR
SBPASE-CHIP-7R	AGGAATTAACACAAGCTGTTCCAC	ChIP-qPCR
SBPASE-qPCR-2F	AAAGAGAAATACACACTGCGATACAC	qRT-PCR
SBPASE-qPCR-2R	TAGGAGAAGTCACATTTGTGAAGATT	qRT-PCR
atalkbh10b-2-LP	AGTAGAAAACACATGCCTCGG	Genotyping
atalkbh10b-2-RP	TTAACATCGAGCCAATTCCAC	Genotyping
atalkbh10b-1-LP	TCCCTCTCATCACCAACAAAG	Genotyping
atalkbh10b-1-RP	ATGCCATAGCCATGAAGATTG	Genotyping
atatx1-LP	AATGAAAGCATGCGGATACAC	Genotyping
atatx1-RP	TCCGTGTTGACTGGAAAGATC	Genotyping

LBb1.3	ATTTTGCCGATTTCGGAAC	Genotyping
