# Supplementary figures and images for: mRNA m6A regulates gene expression via H3K4me3 shift in 5’ UTR
Source: Genome Biol. 2025 Mar 12;26:54. doi: 10.1186/s13059-025-03515-8 (PMC11900566; doi:10.1186/s13059-025-03515-8)

**Fig. 4B**

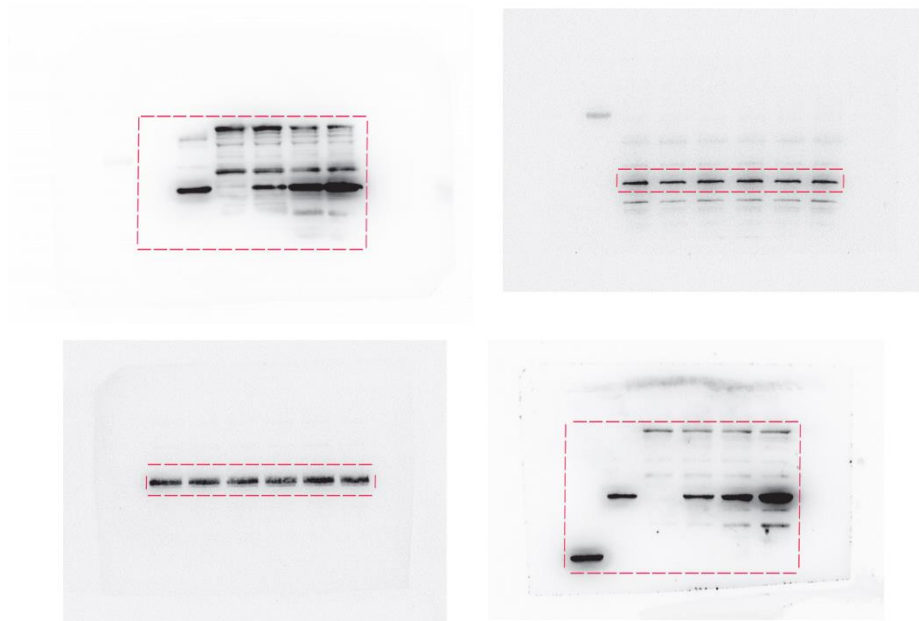

**Fig. 5A**

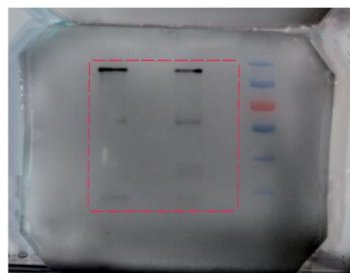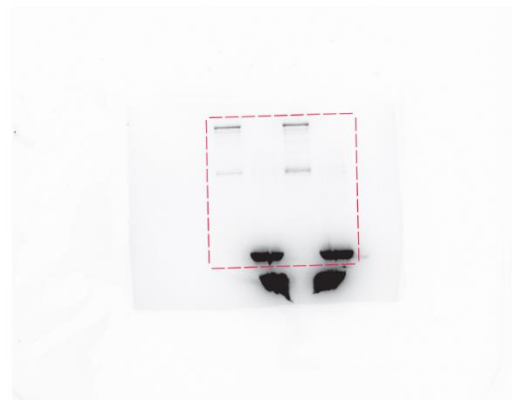

**Fig. 5B**

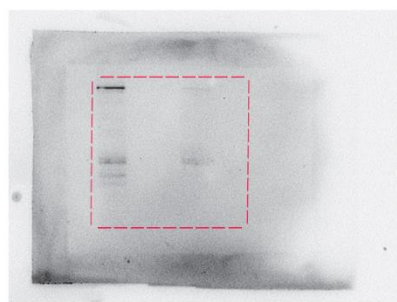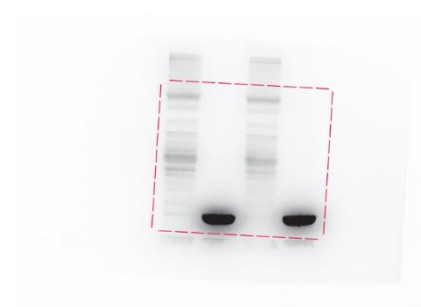

**Fig. 5C**

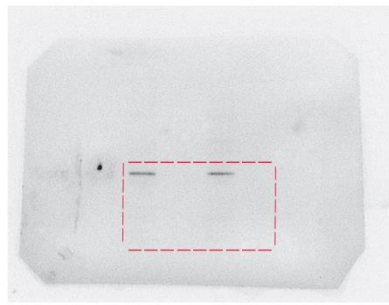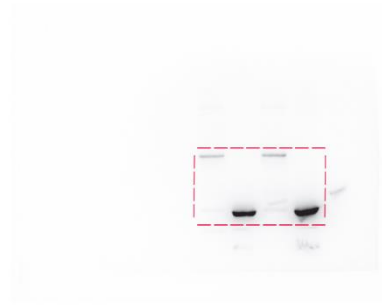

**Fig. 5D**

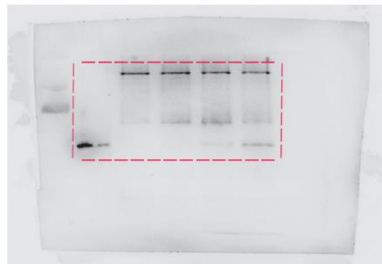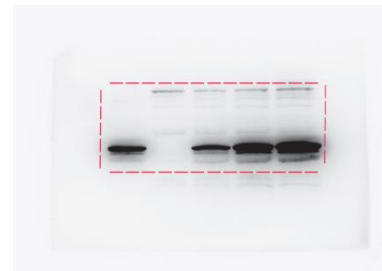

**Fig. 5E**

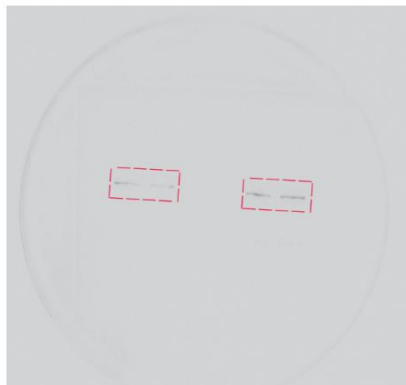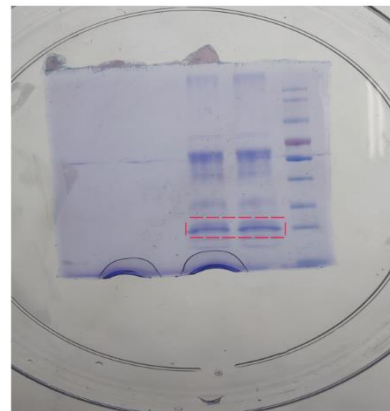

**Fig. 6B**

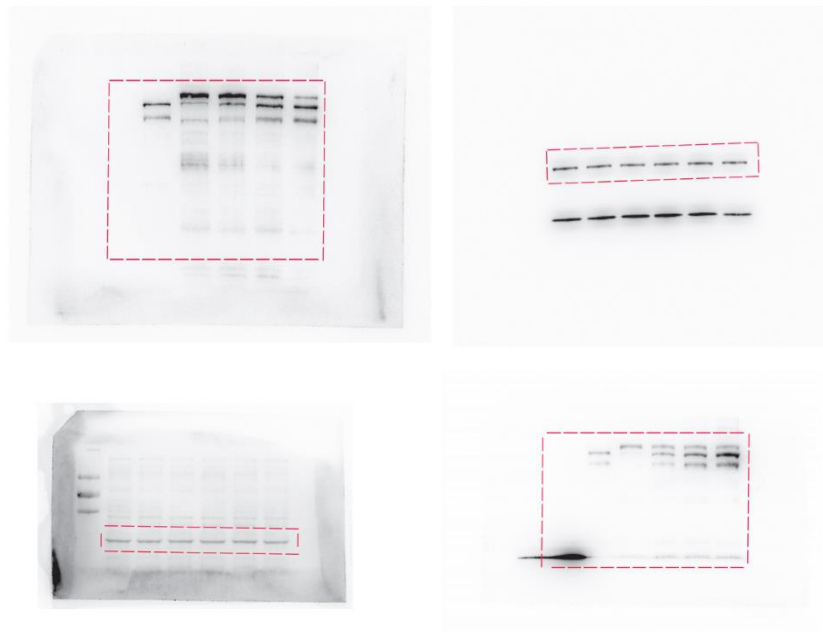

**Fig. 6E**

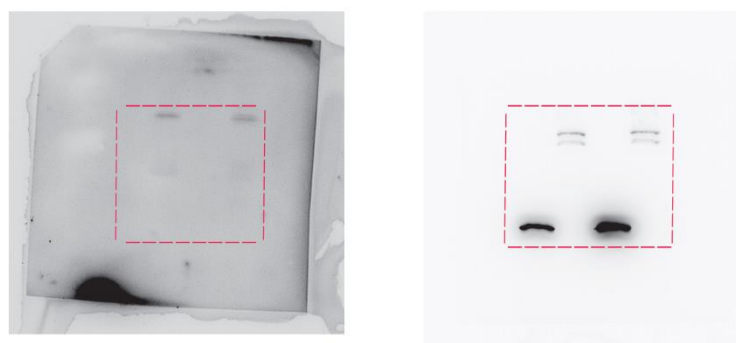

**Fig. 6F**

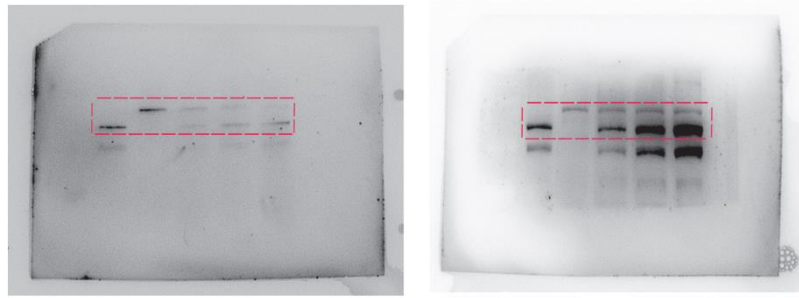

**Fig. 6G**

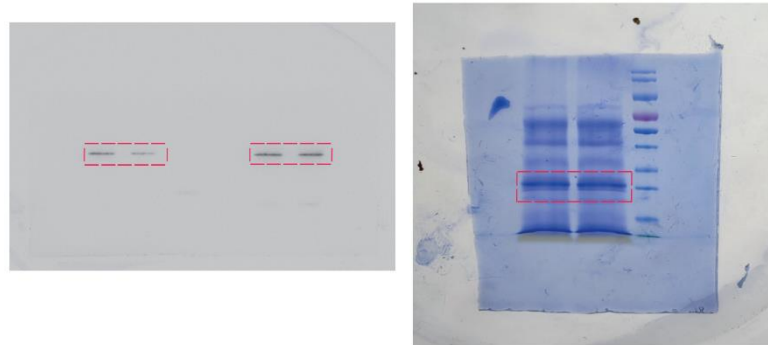

**Fig. S4D**

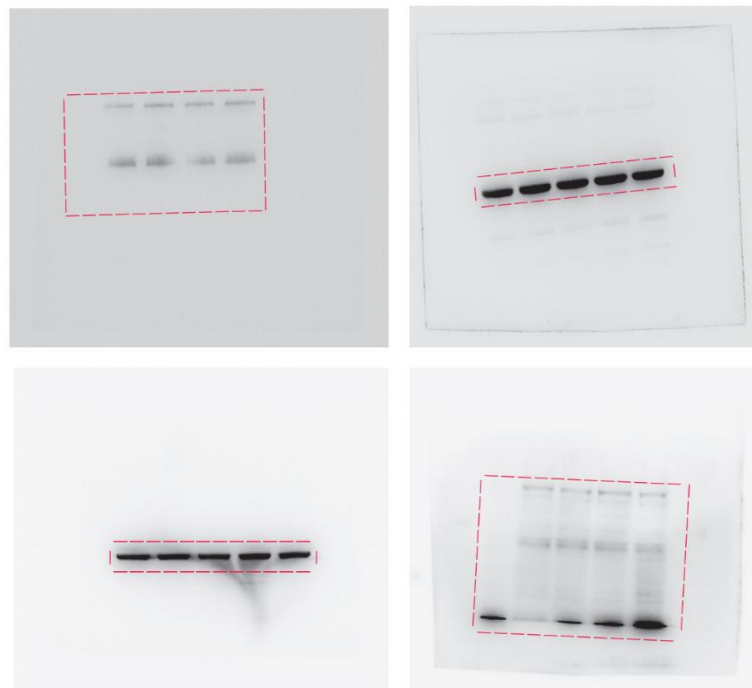

Supplement: Supplementary file 3 — Additional file 3: Uncropped images of Western blots in Fig. 4, Fig. 5, Fig. 6 and Fig. S4 [file 13059_2025_3515_MOESM3_ESM.pdf]
